# The impact of pain on memory: a study in chronic low back pain and migraine patients

**DOI:** 10.1093/braincomms/fcaf486

**Published:** 2025-12-10

**Authors:** Katarina Forkmann, Vanessa C Dobischat, Katharina Schmidt, Katrin Scharmach, Dagny Holle, Katja Wiech, Ulrike Bingel

**Affiliations:** Clinic for Neurology, Center for Translational Neuro- and Behavioral Sciences (C-TNBS), University Hospital Essen, University of Duisburg-Essen, 45147 Essen, Germany; Clinic for Neurology, Center for Translational Neuro- and Behavioral Sciences (C-TNBS), University Hospital Essen, University of Duisburg-Essen, 45147 Essen, Germany; Clinic for Neurology, Center for Translational Neuro- and Behavioral Sciences (C-TNBS), University Hospital Essen, University of Duisburg-Essen, 45147 Essen, Germany; Clinic for Neurology, Center for Translational Neuro- and Behavioral Sciences (C-TNBS), University Hospital Essen, University of Duisburg-Essen, 45147 Essen, Germany; Clinic for Neurology, Centre for Translational Neuro- and Behavioral Sciences (C-TNBS), West German Headache Centre, University Hospital Essen, University of Duisburg-Essen, 45147 Essen, Germany; Clinic for Neurology, Center for Translational Neuro- and Behavioral Sciences (C-TNBS), University Hospital Essen, University of Duisburg-Essen, 45147 Essen, Germany; Wellcome Centre for Integrative Neuroimaging (WIN), Nuffield Department of Clinical Neurosciences, University of Oxford, John Radcliffe Hospital, Oxford OX3 9DU, UK; Clinic for Neurology, Center for Translational Neuro- and Behavioral Sciences (C-TNBS), University Hospital Essen, University of Duisburg-Essen, 45147 Essen, Germany

**Keywords:** pain-cognition interference, expectation, recognition, recollection, familiarity

## Abstract

Patients with chronic pain often complain of cognitive difficulties, such as ‘poor memory’. Both acute and chronic pain are thought to impair cognitive performance by demanding attentional and cognitive resources to the detriment of cognitive functioning. However, systematic experimental investigations in patients, as well as deeper understanding of factors that modulate these effects remain lacking. This study investigated whether patients with chronic migraine or patients with chronic low back pain are more susceptible to the disruptive effects of pain on memory as compared to pain-free healthy controls. Two groups of individuals with chronic pain (*n* = 55 patients with chronic migraine, *n* = 59 patients with chronic back pain) and *n* = 59 age-matched healthy controls, underwent experimental pain stimulation at either the back or head while performing a visual categorization and a subsequent recognition task. Pain-related cognitions and clinical parameters were assessed to explore their influence on pain-cognition interference. This large-scale experimental study revealed encouraging results regarding the impact of experimental pain on memory for the pain disorders studied here. Contrary to our hypothesis, patients with chronic migraine or chronic back pain showed no greater effects of experimental pain on recognition memory than healthy participants. Furthermore, the study showed no effect of stimulation site (i.e. head or lower back) or interaction with type of chronic pain. Pain-related cognitions, psychological variables and clinical parameters only had a marginal effect on pain-induced impairment of recognition memory in pain patients. Future research should focus on identifying cognitive and neural predictors associated with susceptibility or resilience to the disruptive effects of pain. Furthermore, larger and more diverse samples could enable person-centred methods to investigate how cognitive, clinical, and situational factors interact in shaping cognitive performance under pain. Such insights are crucial for the development of targeted, individualized therapeutic approaches in the management of chronic pain syndromes.

## Teaser

Patients with chronic pain often report poor memory. Forkmann *et al*. found that patients with chronic back pain and chronic migraine do not show greater disruptive effects of experimental pain on episodic memory than healthy, pain-free individuals. Clinical and psychological parameters had only minor influence on pain’s effect on memory.

## Graphical Abstract

**Graphical Abstract:**
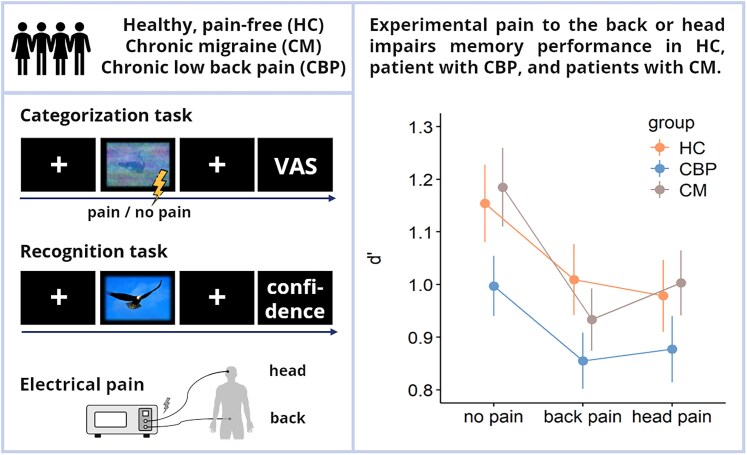

## Introduction

Acute pain serves an inherent warning function. Even in complex, multi-sensory environments, it prompts an attentional shift towards pain, to enable a timely response, such as swiftly moving away from the potential source of danger. Eccleston and Crombez referred to this attention-capturing property of pain, that overrides other cognitive processes as the ‘interruptive function of pain.^1^ In chronic pain, however, the acute source of danger is no longer present, rendering the once-protective warning character of pain physiologically irrelevant. Yet, the interruptive effect remains which may contribute to the frequently reported cognitive impairments in chronic pain patients, including memory deficits.^2-5^ The ongoing impact of pain on cognitive processes can result in significant functional deficits and a reduced quality of life in patients with chronic pain.^6,7^

Several bottom-up and top-down factors have been discussed to determine the degree to which pain affects cognitive processing in the individual including pain intensity,^8-10^ pain-related anxiety^11-13^ and pain catastrophizing.^11,14-17^ Importantly, previous studies investigating pain’s effect on cognition often focused on healthy participants and, to our knowledge, no study was designed to compare different pain conditions with respect to pain-induced task interference.

Experimental pain studies in healthy participants suggest that pain location may influence pain’s effects on cognition, but this remains unexplored in chronic pain. The trigeminal system, in particular, appears to play a critical role, as evidenced by several studies comparing pain stimuli applied to different body sites. For instance, repeated pain stimuli to the face, compared to the hand, led to greater sensitization, likely due to facial pain being perceived as more threatening than pain in other regions, such as the arm.^18^ Similarly, another study using classical conditioning found a stronger propensity to form cue-pain-associations when pain was applied to the face rather than the hand.^19^ Further evidence comes from research on the hand blink reflex—a natural defence response triggered by electrical stimulation of the median nerve—underscoring the heightened relevance of pain near the head and face.^20,21^ Building on these findings, we hypothesize that nociceptive stimulation of the trigeminal system or head interferes more strongly with concurrent cognitive processing compared to the same stimuli delivered to other body regions.

This study investigated the effect of experimentally induced pain on higher-order cognitive functioning in patients with chronic back pain (CBP) or chronic migraine (CM) compared to age-matched healthy participants (HC). Participants completed a visual categorization task, categorizing images of objects as either living or non-living while receiving concurrent electrical pain stimuli to the forehead or lower back. A subsequent recognition task assessed the effect of pain on memory. The study examined whether interference differed by stimulation site or was more pronounced at the site of chronic pain in the two groups with chronic pain. Furthermore, we explored how pain-related cognitions (e.g. fear of pain), expectations of pain-induced task interference, perceived attention impairment as well as affective (anxiety, depression, stress) and clinical parameters (e.g. clinical pain intensity and frequency) modulated the effect of pain on memory.

## Methods

### Participants

Healthy participants and both patient samples were recruited between May 2017 and August 2021 locally from our in-house database and via advertisements in local and social media. Migraine and back pain patients were also recruited via the local back pain clinic (head: UB) and the local headache centre (head: DH) using leaflets and personal approach by the scientific staff. Inclusion criteria for both groups were age 18–80 years, normal or corrected-to-normal vision and written informed consent to participate in the study. Patients were included in the study, if they had a pre-diagnosis of chronic low back pain (duration > 3 months; definition according to the IAPS^22^) or chronic migraine (duration > 3 months and at least 8 migraine and 15 headache days per month; definition according to the ICHD-3^23^). The eligibility of patients was confirmed by physicians specializing in neurology and pain medicine (UB, JKB) using medical records and clinical examination. Healthy control participants were age-matched to the patient groups. Exclusion criteria comprised participation in trials using investigational medicinal products within the last three months, current major psychiatric disorder (e.g. major depression, anxiety disorder), pregnancy or breastfeeding, medication overuse headache (MOH), chronic pain disorder other than chronic low back pain or migraine (e.g. radicular pain, neuropathic pain), use of high-dose opioids (>100 mg morphine equivalent per day). Participants who agreed to additional MR assessment (resting state fMRI and anatomical MRI) were also screened for contraindications to MR assessment (e.g. pacemakers, magnetizable parts, claustrophobia). Contraindications for healthy participants also included chronic pain and regular medication (especially analgesics).

The required sample size was calculated based on previous studies investigating memory performance in migraine patients^24^ and results from studies investigating interference from acute experimental pain in healthy participants.^11,25^ We used GPower 3.0 to calculate the required minimum sample size with parameters *f* = 0.20, *α* = 0.05, power (1-*β*) = 0.8, resulting in a sample size of 50 participants per group. To allow for a 20% attrition rate, 60 participants per group need to be recruited and tested.

A total of 181 participants were included in the study (*n* = 60 HC, *n* = 61 CM, *n* = 60 CBP). After exclusion, data of *n* = 173 participants were analysed. Reasons for exclusion were as follows: CBP: inability to understand the encoding task (*n* = 1), HC: high level of pain tolerance resulting in unsuccessful calibration (*n* = 1), CM: data loss due to technical reasons (*n* = 2), presence of episodic rather than chronic migraine (*n* = 1), presence of additional back pain and irritable bowel syndrome (*n* = 1) and cognitive deficits resulting in test termination (*n* = 1). The sample description of the analysed data set is given in Table 1 and Supplementary Table 1.

**Table 1:** Sample description: demographic and clinical variables (descriptives and group comparisons)

	Group			Inference
Variable	HC	CBP	CM	
*N*	59	59	55	
Age	40.0 ± 15.2	43.6 ± 16.6	38.7 ± 14.4	*χ²*(2) = 2.43, *P* = 0.30
Gender	23 male, 36 female	24 male, 35 female	9 male, 46 female	*χ²(2)* *=* *9.48,* *P* *=* 0.009*
Medication use	Others (regular): *n* = 14	Antidepressants: *n* = 7 NSAID: *n* = 27 (7 regular) Non-opioid analgesics: *n* = 10 (2 regular) Opioid analgesics: *n* = 3 (1 regular) Pregabalin/Gabapentin: *n* = 3 Others: *n* = 26	*Prophylaxis:* Antidepressants: *n* = 13 Antiepileptics: *n* = 2 Anticonvulsants: *n* = 2 Beta-blocker: *n* = 5 Botox: *n* = 3 CGRP-Antagonists: *n* = 2 *Acute pain medication:* Non-opioid analgesics: *n* = 12 NSAID: *n* = 29 Opioid analgesics: *n* = 1 Triptans: *n* = 35 Others: *n* = 28	
Aura				
Yes	-	-	29 (52.7%)	
No			20 (36.4%)	
Missing			6 (11.8%)	
German Pain Questionnaire				
Current pain level [NRS 0–10]	-	2.86 ± 2.05 (*n* = 58)	3.07 ± 2.94 (*n* = 54)	*W* = 1607.5, *P* = 0.81^§^
Maximum pain level, last 4 weeks [NRS 0–10]	-	6.79 ± 1.90 (*n* = 58)	8.33 ± 1.26 (*n* = 54)	W = 795.5, *P* < 0.001*^§^
Average pain level, last 4 weeks [NRS 0–10]	-	4.33 ± 1.64 (*n* = 58)	5.89 ± 1.73 (*n* = 54)	W = 848, *P* < 0.001*^§^
Von Korff grade				
1	-	29 (49.1%)	16 (29.1%)	
2	-	15 (25.4%)	13 (23.6%)	
3	-	12 (20.3%)	20 (36.4%)	
4	-	2 (3.4%)	5 (9.1%)	
NA	-	1 (1.7%)	1 (1.8%)	
Chronic pain present since	1–6 months	1 (1.7%)	0 (0%)	
	6–12 months	4 (6.8%)	2 (3.6%)	
	1–2 years	7 (11.9%)	1 (1.8%)	
	2–5 years	8 (13.6%)	5 (9.1%)	
	> 5 years	38 (64.4%)	46 (83.6%)	
	NA	1 (1.7%)	1 (1.8%)	
Number of pain days, last 4 weeks	0.17 ± 0.58 (*n* = 12)	19.90 ± 9.06 (*n* = 58)	15.05 ± 6.96 (*n* = 54)	W = 2078.5, *P* = 0.003*^§^
Number of pain medication days, last 4 weeks	0.20 ± 0.63 (*n* = 10)	8.95 ± 11.49 (*n* = 57)	9.04 ± 5.75 (*n* = 52)	W = 1035.5, *P* = 0.006*^§^

CBP, patients with chronic lower back pain; CGRP, Calcitonin gene-related peptide; CM, Patients with chronic migraine; HC, healthy controls; NA, not available; NRS, numeric rating scale; NSAID, non-steroidal anti-inflammatory drug.

^*^ *P* < 0.05. ^§^ Statistical comparison of CM and CBP group.

The study was approved by the local Ethics Committee (University of Duisburg-Essen, Germany; 15-6683-BO) and was conducted in accordance with the Declaration of Helsinki. The study protocol was registered with the German Clinical Trials Register (DRKS00012448). All participants gave written informed consent and received monetary compensation for their participation. They were free to withdraw from the study at any time without any consequences, particularly concerning their ongoing medical care.

### Experimental design and procedures

The study was performed on 2 days (1–5 days apart). A third day, on which the MR assessment was performed, was optional and is not reported here.

On the first day, questionnaires were completed to assess demographic and clinical data as well as pain-related personality traits (e.g. pain catastrophizing, pain anxiety), and a battery of neuropsychological tests was administered. The subsequent preparatory procedures involved the assessment of pain thresholds at the face and lower back, and the calibration and matching of back pain and head pain stimuli within each individual. To test whether the matching procedure was successful, three pain stimuli were applied to each stimulation site and participants were asked to rate the pain intensity using a 0–100 visual analogue scale (VAS, see below). Before the memory task began, participants answered two questions to assess their fear of pain and expectation of pain-cognition interaction.

#### Assessment of clinical parameters

All patients completed the German Pain Questionnaire (GPQ^26^), a diagnostic tool that assesses pain characteristics such as type, location, intensity, duration and medication use. The GPQ grades chronic pain severity (grades 1–4) based on pain intensity and pain-related disability and evaluates factors like depression, anxiety and stress. Additionally, participants completed a brief questionnaire on the number of pain days and days of pain medication use in the past month.

#### Questionnaires and neuropsychological testing

To assess group differences and their potential influence on pain-induced interference, participants completed the German version of the following questionnaires: (i) Pain Anxiety Symptom Scale (PASS-D^27,28^); (ii) Pain Catastrophizing Scale (PCS^29,30^); (iii) Depression Anxiety Stress Scale (DASS-21^31,32^), (iv) State Trait Anxiety Inventory (STAI trait scale^33,34^) and (v) Questionnaire of Experienced Attention Deficits (FEDA,^35^ subscales: distractibility and slowing down in mental processes (FEDA-AV), fatigue and slowing down in practical activities (FEDA-EV), reduction in motivation (FEDA-AM). Migraine patients also completed the Headache-Impact-Test (HIT-6^36^) to evaluate the impact of headache on daily functioning and well-being.

Cognitive functioning was assessed using a comprehensive neuropsychological test battery to identify group differences in baseline cognitive performance that could explain variations in pain-cognition interference during the experimental task. The test and subtests used are detailed in Supplementary Table 2. All questionnaires and neuropsychological tests were analysed according to their manuals, using age-, gender- and education-adjusted values (e.g. T-values, percentile ranks) where available to account for the heterogeneity of the study sample.

#### Assessment of pain thresholds and pain stimuli calibration

To determine pain thresholds at the stimulus application sites, single electrical pulses (0.5 s duration) were delivered, starting at 0 mA and increasing in 0.1 mA increments until participants indicated a painful sensation (instructions adapted from^37^). This procedure was repeated three times per stimulation site. The pain threshold was calculated as the average of the reported intensities for each site. The order of stimulation sites was counterbalances across individuals.

To determine pain stimuli corresponding to a VAS score of 70, trains of electrical pulses (total duration: 2.5 s) were delivered with increasing intensity (starting at 0.0 mA, steps of 0.5 mA, ISI approx. 2–3 s). Participants rated the pain intensity (‘How painful was this stimulus?’) on a VAS ranging from 0 (‘not painful at all’) to 100 (‘unbearably painful’) by adjusting a red bar between the endpoints using two buttons operated with their index and middle fingers. The stimulus intensity was increased until the participant reported a VAS score of 70, which was then confirmed by presenting the same stimulus intensity twice. This procedure was repeated for the second stimulation site, with the order of sites counterbalanced across participants.

#### Assessment of fear of pain and expectation

To examine the influence of subjective fear and expectation of the upcoming painful stimulation on the disruptive effects of experimentally induced pain, participants answered two questions before the experiment. Fear of pain was rated separately for each stimulation site using a 0–100 VAS with the question: ‘How fearful are you regarding the upcoming pain stimulation on the head?’ and ‘How fearful are you regarding the upcoming painful stimulation on the back?’ (VAS anchors: 0 = ‘not fearful at all’, 100 = ‘extremely fearful’). Additionally, participants rated their expectations regarding the impact of pain on task performance using the question: ‘How do you expect pain to influence your task performance?’ (VAS anchors: −50 = ‘strong performance decrease’, 0 = ‘no influence’, 50 = ‘strong performance increase’). Expectation was not assessed separately for each stimulation site.

#### Encoding task and recognition task

To quantify the interruptive effect of pain, all participants completed a categorization (encoding) and subsequent recognition task as described previously.^11,25,38^ In the categorization task, images had to be categorized into living or non-living objects. The categorization task (duration: approximately 16 min) began with 6 practice trials (2 trials per condition: 3 living and 3 non-living objects) presented in random order. Following this practice period, 60 images (30 living, 30 non-living objects) with reduced visibility were presented under three conditions: (i) concurrent electrical stimulation applied to the lower back (*back pain*, 20 trials), (ii) concurrent electrical stimulation applied to the forehead (*head pain*, 20 trials) or (iii) no painful stimulation (*no pain*, 20 trials). Thus, 40 painful electrical stimuli were applied during the encoding task. Importantly, the *head pain* and *back pain* stimuli were matched intra-individually for pain intensity. Each condition comprised 10 living and 10 non-living objects and conditions were presented in a pseudo-randomized order, with no more than three consecutive trials of the same type.

Categorization accuracy (living/non-living), response times (RT, in ms) and pain intensity ratings (0–100 VAS) were recorded as outcome variables.

To quantify the effect of *head pain* and *back pain* on memory encoding, a surprise recognition phase followed the categorization task (lasting approximately 14 min). In this phase, all 60 images from the categorization task were presented alongside 60 new images, resulting in a total of 120 images. Participants indicated whether each image was ‘old’ (previously seen) or ‘new’ (unseen) immediately after its presentation, using a 6-point confidence scale (anchors ‘definitely old’ to ‘definitely new’).

**Figure 1:**
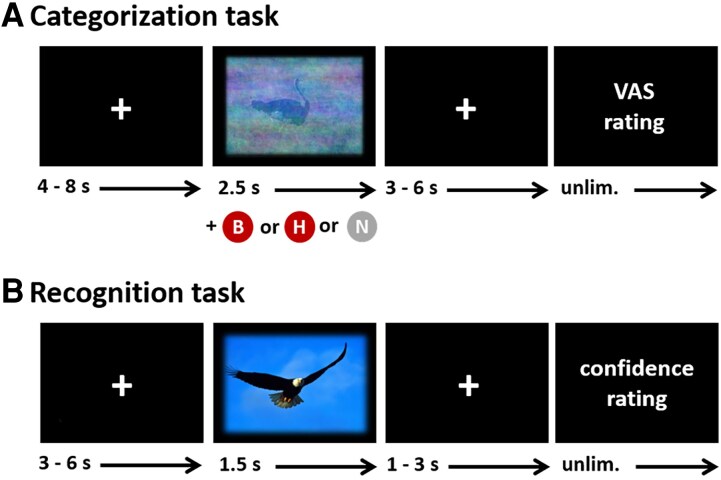
**Experimental paradigm.** (**A**) Categorization task. Trial structure: A white fixation cross was displayed for 4–8 s, followed by the simultaneous presentation of a reduced visibility image with *back pain* (B), *head pain* (H) or *no pain* (NP) for 2.5 s. This was followed by another white fixation cross (3–6 s) and a pain intensity rating (no time limit). Participants were instructed to categorize the images as living or non-living as quickly as possible using two keyboard keys (left/right arrow; category assignment counterbalanced across participants). At the end of each trial, participants rated the pain intensity (‘How painful was this stimulus?’) using a 0–100 VAS (verbal anchors: 0 = ‘not painful at all’ and 100 = ‘unbearably painful’). **(B)** Recognition task. Trial structure: white fixation cross (3–6 s), presentation of new or old image (1.5 s), white fixation cross (1–3 s), confidence rating (no time limit). B, back pain; H, head pain; NP, no pain; s, seconds; unlim., unlimited; VAS, visual analogue scale.

Confidence ratings for each image were recorded as the outcome measure. For details regarding the paradigm and trial structure, see Fig. 1.

### Stimuli

#### Electrical stimuli

Painful electrical stimulation was applied to the lower back (at the level of segments L3–L5) and the forehead (V1 supply area) using two identical constant current stimulators (Digitimer DS7A, Hertfordshire, UK) and surface electrodes (Specialty Developments, Bexley, UK) with a diameter of approximately 5 mm, secured with medical tape. Each 2.5 s pain stimulus consisted of single pulses (0.5 ms duration) delivered at 30 ms intervals. The stimulation was controlled using Presentation software (Neurobehavioral Systems, Inc., Berkeley, CA, www.neurobs.com).

#### Visual stimuli

The visual stimuli consisted of pictures showing natural scenes with living or non-living objects of neutral valence.^11^ A total of 120 images were selected, with 60 from each category. During the categorization task, 30 images from each category were presented with reduced visibility,^11^ while all 120 images (full visibility) were included in the recognition task. The stimuli were displayed on a computer monitor, positioned approximately 60 cm from the participants, with each image having a visual angle of 13.3°×9.5° and a display duration of 2.5 s.

### Statistical analysis

All behavioural data were automatically recorded by the software Presentation and analysed using R version 4.0.2.^39^

Descriptive data are presented as mean and standard deviation (M ± SD), unless stated otherwise. Group differences in pain-related variables (electrical pain thresholds, stimulus intensity corresponding to VAS70, mean pain intensity ratings during the encoding task, site-specific fear of pain and expectations of disruptive effects of pain) and person-related variables (questionnaire and neuropsychological data) were analysed using parametric (*t*-test, ANOVA) or non-parametric tests (Mann–Whitney *U*; Kruskall–Wallis), as appropriate. Significant results of these exploratory analyses were followed by either Bonferroni-corrected two-tailed post-hoc t-tests or Wilcoxon-rank sum tests. Results with *P* < 0.05 are considered significant and effect sizes (*η_p_²* and Cohen’s *d*) are reported.

#### Categorization task

Categorization performance was indexed by the *percentage of correct classifications* per condition and the *mean reaction times* (*RT*) for correctly categorized images only, separately for each experimental condition. Before calculating the individual mean RT, trials with RT <200 or >2,500 ms, and trials with unusually deviant RT were discarded (individual cutoff per participant: ±3 SD). Mean *pain intensity ratings* were calculated separately for each experimental condition.

#### Recognition task

The detrimental effect of pain on memory was assessed using the discrimination index *d’*^40^ and the parameters *recollection* and *familiarity,* which, according to the dual-process model of memory,^41^ represent two distinct memory retrieval processes underlying recognition memory. While *recollection* refers to high-confidence remembering of an item along with contextual details (e.g. thoughts or feelings during encoding), *familiarity* reflects a sense of knowing an item without recalling specific context. Unlike *recollection*, *familiarity* can span a range of confidence levels. To calculate *d’*, confidence ratings were dichotomized (confidence ratings 1–3 = *old*; 4–6 = *new*) and the individual hit rate (i.e. correctly identified old images) and false alarm rate (i.e. incorrectly identified new images) were calculated. The index *d’* was computed separately for each condition using the formula: *d’*  *=*  *z(hit rate)—z(false alarm rate)* with higher *d’* values indicating better recognition memory. Note that *d’* of one CM patient required transformation prior to further calculations due to perfect accuracy in the no pain condition (i.e. infinite *d’*).^42^ Parameter estimates for the outcome variables *recollection* and *familiarity* were calculated separately for each condition using maximum likelihood estimation,^43^ applying the dual-process signal detection model^41,44^ to individual confidence ratings.

To assess differential effects on each outcome variable between groups and conditions, linear mixed model (LMM) analyses were conducted using the R package *lme4.*^45^ Separate models were calculated for each outcome variable (*encoding*: percent correct categorization, mean RT; *recognition*: d’, familiarity, recollection). To examine the disruptive effect of experimental pain and whether it differed between patients and age-matched healthy controls, the LMMs included fixed effects for the factors *condition* (back pain, head pain, no pain) and *group* (HC, CM, CBP). To investigate site-specific effects (i.e. a stronger interruptive effect of pain at the back in the CBP group or a stronger interruptive effect of head pain in the CM group), we computed restricted LMMs for the two patient groups only, focusing on the differences between no pain and each pain condition (i.e. *d’ (no pain)—d’ (back pain)* and *d’ (no pain)—d’ (head pain)*). In the case of significant two-way or three-way interactions, additional LMMs were performed separately for each group. Significant effects were followed by Bonferroni-corrected post-hoc tests using the R package *emmeans.*^46^

As we were particularly interested whether pain-related cognitions and disease-related parameters would modulate the effect of experimental pain on recognition memory in patients with chronic pain, the following variables were separately included as potential covariates in the different LMMs (outcome parameters: *d’*, *recollection*, *familiarity*) for exploratory analyses: pain catastrophizing (PCS), pain anxiety (PASS), anxiety, stress and depression (DASS), fear of experimentally induced pain (assessed separately for each stimulus site), expectation of pain-task interference (assessed irrespective of the site of painful stimulation), experienced attention impairments in everyday life (FEDA subscales), perceived migraine-related disabilities (HIT-6, CM group only), current clinical pain intensity (GPQ), average pain intensity during the last 4 weeks before study participation (GPQ), number of pain days during the last 4 weeks before study participation and disease severity (von Korff grade, GPQ).

All models were estimated using the restricted maximum likelihood approach, with the best model selected based on the Akaike information criterion (maximum likelihood approach) and significance determined by a χ²-test for model comparison. If a model including a potential covariate did not significantly improve over a model without it, the covariate was considered to have no influence on the effects of the fixed factors *group* and *condition*.

## Results

### Demographics and pain-related data

Groups were comparable in age (χ²(2) = 2.43, *P* = 0.30) but differed significantly in gender distribution (χ²(2) = 9.47, *P* = 0.009). The number of female participants was significantly higher in the CM group (Table 1), in line with the higher prevalence of migraine in women.^47^ Most patients (73.7%) had experienced pain for more than 5 years.

On both study days significantly more CBP patients (81.4%) than CM patients (60.0%) reported having pain at the start of the examination. However, pain intensity of the patients’ reporting pain was comparable in both patient groups. See Table 1 and Supplementary Table 1 for a more detailed sample description regarding different clinical pain variables.

### Questionnaire data

Significant group differences were found across all questionnaires (Supplementary Table 3). Post-hoc comparisons revealed that both patient groups scored significantly higher than the HC group in depression, anxiety and stress (DASS), pain catastrophizing (PCS) and pain-related anxiety (PASS). Further, both patient groups reported significantly stronger experienced attention impairments in everyday life (FEDA). Significant differences between the two patient groups were only found for pain-related anxiety (PASS) and anxiety and stress (DASS), which were higher in CM than in CBP patients.

### Neuropsychological data

Extensive testing across cognitive domains revealed that the groups differed in two of the 17 evaluated parameters, namely verbal flexibility (RWT, alternating listing of items from two categories): χ²(2) = 8.51, *P* = 0.01) and divided attention (TAP, number of missed signals): χ²(2) = 9.37, *P* = 0.009). Post-hoc group comparisons showed that CM performed significantly worse on verbal flexibility compared to HC (*W* = 1174, *P* = 0.03) and CBP (*W* = 2068, *P* = 0.03) and that CBP showed lower performance in divided attention as compared to HC (*W* = 1140, *P* = 0.006). Descriptive data and inference statistics are provided in Supplementary Table 4.

### Expectation and pain-related fear

All three groups expected experimentally induced pain to significantly impair task performance (HC: *V* = 128.5, *P* < 0.001; CBP: *t*(57) = −4.58, *P* < 0.001; CM: *t*(54) = −6.94, *P* < 0.001). However, the expectation of pain-task interference did not significantly differ between groups (*F*(2,169) = 1.37, *P* = 0.26). Exploratory correlation analyses showed that the expectation of pain-task interference did not scale with disease parameters (e.g. current and average clinical pain intensity, disease severity) or psychological and pain-related variables (e.g. anxiety, stress, depression, pain catastrophizing, pain-related anxiety; see Supplementary Table 5 for results).

Fear ratings provided prior to the pain experiment were significantly higher for head pain compared to back pain (main effect [ME] *condition* (F(1,168.20) = 10.98, *P* = 0.001). The *group × condition* interaction did not reach significance (*F*(2,168.20) = 2.72, *P* = 0.07). Exploratory post-hoc tests showed that fear ratings did not significantly differ between HC and CBP for both stimulation sites (HC: *t*(168) = −1.25, *P* = 0.21; CBP: *t*(169) = −0.70, *P* = 0.49), whereas CM reported significantly higher fear ratings for head pain than for back pain (*t*(168) = 3.75, *P* < 0.001). For descriptive data, see Table 2.

**Table 2:** Ratings of expected pain-task interference, fear of pain and ratings related to painful stimulation (M ± SD)

	Group		
Variable	HC	CBP	CM
Fear of pain [VAS 0–100]			
back	25.12 ± 25.02	23.74 ± 24.38 (*n* = 58)	22.71 ± 22.19
head	28.34 ± 26.37	25.23 ± 23.47 (*n* = 57)	32.69 ± 27.08
Expectation [VAS −50–50]^a^			
Expectation of pain-task interference	−16.00 ± 16.88	−11.00 ± 18.30 (*n* = 58)	−14.82 ± 15.84
Pain thresholds (mA)			
back	1.80 ± 1.65	1.57 ± 1.74 (*n* = 58)	1.59 ± 1.51
head	1.40 ± 1.90	1.11 ± 1.07 (*n* = 58)	1.22 ± 1.10
Stimulus intensity (mA) during experiment (corresponding to VAS 70)^b^			
back	2.20 ± 2.39	1.96 ± 2.19	1.68 ± 1.35
head	1.85 ± 2.27	1.74 ± 1.97	1.59 ± 1.68
Pain intensity rating during the experiment [VAS 0–100]^c^			
back	61.99 ± 10.87	60.03 ± 12.99	62.62 ± 9.44
head	61.49 ± 9.18	59.03 ± 12.41	64.45 ± 7.5

CBP, patients with chronic lower back pain; CM, Patients with chronic migraine; HC, healthy controls; mA, milliampere; VAS, visual analogue scale.

^a^negative values correspond to an expectation that pain will negatively affect task performance.

^**b**^see Supplementary Table 8 for stimulus intensities applied during the categorization task (first and final stimulus intensity).

^c^see Supplementary Analyses for changes in pain intensity ratings throughout the categorization task.

### Pain thresholds, pain stimuli and pain intensity ratings

Pain thresholds and stimulus intensities corresponding to VAS70 were similar across groups but significantly lower for head pain compared to back pain. Pain intensity ratings during the encoding task were comparable across conditions and groups, confirming successful calibration. For descriptive data and statistical inference, refer to Table 2, Supplementary Tables 6 and 8.

### Encoding

#### Hits

Performance in correctly categorizing images as living or non-living did not differ significantly between the three groups (HC, CBP, CM) or the three experimental conditions no pain, head pain and back pain (ME *group*: *F*(2,170) = 2.21, *P* = 0.11, *η²* = 0.03; ME *condition*: *F*(2,340) = 1.70, *P* = 0.18, *η²* = 0.01; IA *group* × *condition*: *F*(4,340) = 0.13, *P* = 0.97, *η²* = 0.002; Supplementary Fig. 1A), indicating that pain did not affect categorization accuracy. For descriptive data see Table 3.

**Table 3:** Categorization and recognition performance separately for each group and each experimental condition (M ± SD)

	Group		
Variable	HC	CBP	CM
Encoding Task			
% correct			
no pain	87.6 ± 14.6	86.7 ± 16.4	92.0 ± 9.30
head pain	88.2 ± 14.4	87.5 ± 16.1	92.4 ± 9.40
back pain	89.2 ± 16.4	88.1 ± 14.9	92.5 ± 9.81
mean RT			
no pain	1167 ± 261	1196 ± 256	1118 ± 244
head pain	1093 ± 234	1157 ± 265	1057 ± 219
back pain	1080 ± 217	1162 ± 274	1037 ± 227
Recognition Task			
d’			
no pain	1.15 ± 0.56	1.00 ± 0.44	1.18 ± 0.55
head pain	0.98 ± 0.52	0.88 ± 0.48	1.00 ± 0.46
back pain	1.01 ± 0.52	0.86 ± 0.41	0.93 ± 0.44
recollection			
no pain	0.19 ± 0.20	0.20 ± 0.19	0.21 ± 0.2
head pain	0.16 ± 0.16	0.19 ± 0.18	0.23 ± 0.21
back pain	0.15 ± 0.14	0.15 ± 0.15	0.17 ± 0.17
familiarity			
no pain	0.87 ± 0.56	0.69 ± 0.42	0.86 ± 0.63
head pain	0.71 ± 0.44	0.60 ± 0.46	0.63 ± 0.44
back pain	0.77 ± 0.51	0.63 ± 0.41	0.68 ± 0.39

CBP, patients with chronic lower back pain; CM, Patients with chronic migraine; HC, healthy controls.

As we were particularly interested in comparing the two patient groups regarding site-specific effects of pain, we performed an analysis including only patients. This analysis revealed no significant main or interaction effects (ME *group: F*(1,112) = 0.34, *P* = 0.56, *η²_p_* = 0.003; ME *condition: F*(1,112) = 0.28, *P* = 0.60, *η²_p_* = 0.002; IA *group × condition*: *F*(1,112) = 0.08, *P* = 0.78, *η²_p_* = 0.001), indicating, that pain did not affect categorization performance in either patient group or condition.

#### Reaction times

Mean reaction times for correctly categorizing images differed significantly between the three conditions (ME *condition*: *F*(2,340) = 27.75, *P* < 0.001, *η²_p_* = 0.14) and marginally between the three groups (ME *group*: *F*(2,170) = 2.69, *P* = 0.07, *η²_p_* = 0.03) while the interaction was not significant (IA *group* × *condition*: *F*(4,340) = 1.61, *P* = 0.17, *η²_p_* = 0.02; see Table 3 for descriptives; Supplementary Fig. 1B). Post-hoc t-tests showed that RTs for head pain and back pain were significantly faster than for the no pain condition (*head pain* versus *no pain*: estimated marginal mean ± SE = −57.95 ± 9.81, *t*(340) = −5.91, *P* < 0.001, *d* = −0.64; *back pain* versus *no pain*: −67.56 ± 9.81, *t*(340) = −6.89, *P* < 0.001, *d* = 0.75), whereas head pain and back pain did not differ significantly (9.61 ± 9.81, *t*(340) = 0.98), *P* = 0.98, *d* = 0.11).

To test for a site-specific effect of pain on RTs in patients, the LMM was restricted to both patient groups. Again, the effects of pain did not significantly differ between patient groups and conditions and no site-specific effect was observed, as indicated by non-significant main and interaction effects (ME *group: F*(1,112) = 2.15, *P* = 0.15, *η²_p_* = 0.02; ME *condition*: *F*(1,112) = 0.62, *P* = 0.43, *η²_p_* = 0.006; IA *group × condition: F*(1,112) = 1.71, *P* = 0.19, *η²_p_* = 0.02).

### Recognition

The disruptive effect of pain on recognition performance was quantified using the parameter *d’* and the memory indices *familiarity* and *recollection.*^41^ Descriptive data are given in Table 3.

#### D’

**Figure 2:**
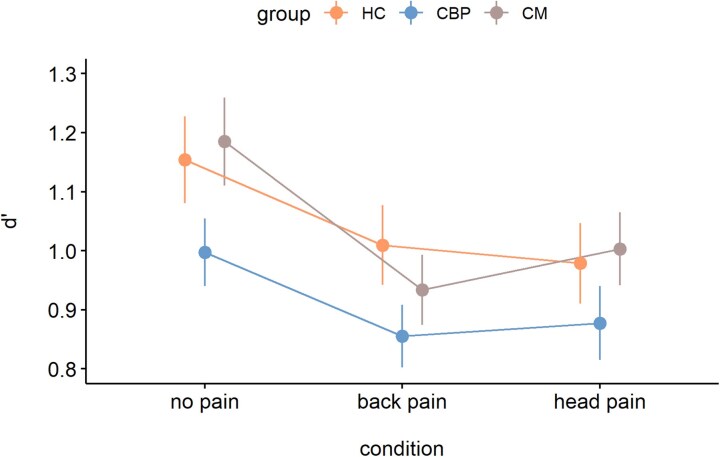
**Experimentally induced pain impairs recognition performance (d’) in patients with chronic pain and healthy participants.** Mean d’ values are shown for the three experimental conditions (no pain, back pain, head pain) and separately for each group (HC, *n* = 59; CBP, *n* = 59; CM, *n* = 55). Error bars indicate the standard error of the mean. Data were statistically analysed using a linear mixed model, which yielded a significant main effect for the factor *condition* (F(2,340) = 18.35, *P* < 0.001; post hoc testing: no pain versus head pain, t(340) = 4.91, *P* < 0.001; no pain versus back pain, t(340) = 5.53, *P* < 0.001; head pain versus back pain, t(340) = 0.62, *P* = 1.00). The main effect of *group* (F(2,170) = 1.96, *P* = 0.14) and the *group × condition* interaction (F(4,340) = 0.79, *P* = 0.53) were not significant. CBP, patients with chronic lower back pain; CM, Patients with chronic migraine; HC, healthy controls.

The LMM including all groups and the three experimental conditions revealed a significant ME *condition* (*F*(2,340) = 18.35, *P* < 0.001, *η²_p_* = 0.10), but no ME *group* (*F*(2,170) = 1.96, *P* = 0.14, *η²_p_* = 0.02) and no *group × condition* interaction (*F*(4,340) = 0.79, *P* = 0.53, *η²_p_* = 0.01). Post-hoc tests showed that *d’* was higher for *no pain* than *head pain* (0.16 ± 0.03, *t*(340) = 4.91, *P* < 0.001, *d* = 0.53) and *back pain* (0.18 ± 0.03, *t*(340) = 5.53, *P* < 0.001, *d* = 0.60), while *head pain* and *back pain* did not differ significantly (0.02 ± 0.03, *t*(340) = 0.62, *P* = 1.00, *d* = 0.07). These results indicate that experimentally induced pain interfered with memory formation, as reflected in significantly lower *d’* values for pain images than no pain images across all three groups (Fig. 2; variability of the effects of pain on recognition memory (d’) is shown in Supplementary Fig. 2).

Again, we compared the two patient groups with respect to the stimulation site. The LMM restricted to patients showed that neither the ME of *group* (*F*(1,112) = 1.64, *P* = 0.20, *η²_p_* = 0.01) and *condition* (*F*(1,112) = 1.40, *P* = 0.24, *η²_p_* = 0.01) nor the *group × condition* interaction (*F*(1,112) = 0.38, *P* = 0.54, *η²_p_* = 0.003) were significant. Thus, both patient groups showed comparable effects of pain on recognition performance and importantly, CM patients did not exhibit greater disruption in the *head pain* condition (relative to the *back pain* condition) than CBP patients showed in the *back pain* condition (relative to the *head pain* condition).

#### Familiarity and recollection

The effects of pain were further tested separately for the memory parameters *familiarity* and *recollection*^41^ (see Table 3 for descriptive data).

**Figure 3:**
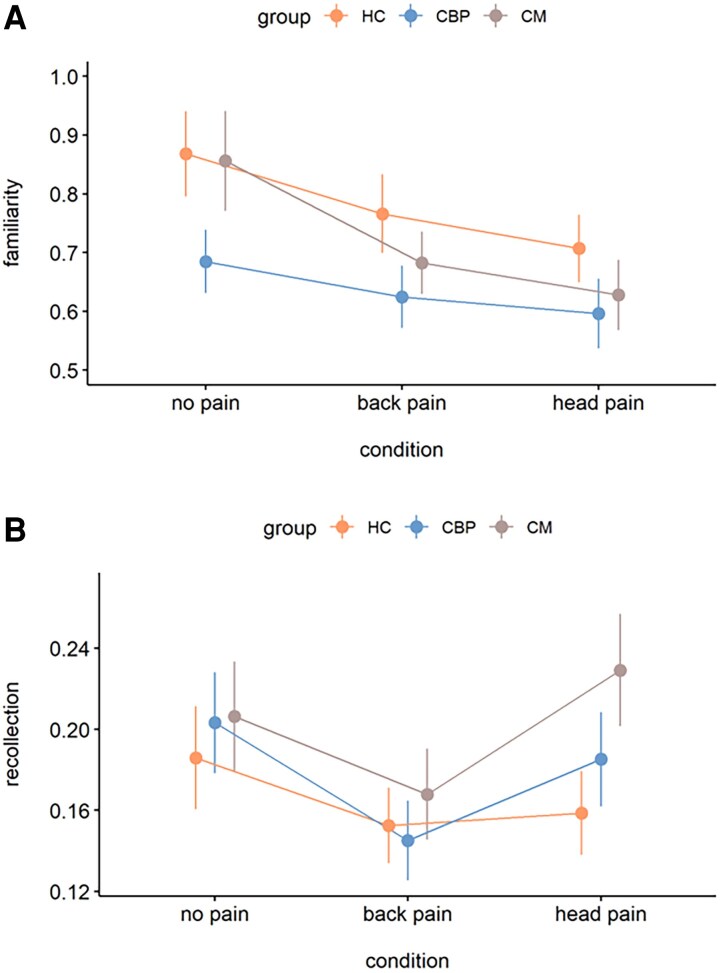
**The effects of experimentally induced back pain and head pain on familiarity-based and recollection-based memory performance.** Mean familiarity (**A**) and recollection (**B**) parameters are shown for the three experimental conditions (no pain, back pain, head pain) and for each group (HC, *n* = 59; CBP, *n* = 59; CM, *n* = 55) separately. Both, back pain and head pain reduced familiarity-based memory but only back pain negatively affected recollection-based memory. Data were statistically analysed using linear mixed models. For familiarity, analyses yielded a significant main effect for the factor *condition* (*F*(2,340) = 8.81, *P* < 0.001; post hoc testing: no pain versus head pain, *t*(340) = 4.09, *P* < 0.001; no pain versus back pain, *t*(340) = 2.87, *P* = 0.01; head pain versus back pain, *t*(340) = −1.22, *P* = 0.67). The main effect of *group* (*F*(2,170) = 2.23, *P* = 0.11) and the *group × condition interaction* (*F*(4,340) = 0.61, *P* = 0.65) were not significant. For recollection, analyses yielded a significant main effect for the factor *condition* (*F*(2,340) = 4.52, *P* = 0.01; post hoc testing: no pain versus head pain, *t*(340) = 0.49, *P* = 1.00; no pain versus back pain, *t*(340) = 2.81, *P* = 0.02; head pain versus back pain, *t*(340) = 2.33, *P* = 0.06). The main effect of *group* (*F*(2,170) = 1.03, *P* = 0.36) and the *group × condition* interaction (*F*(4,340) = 0.76, *P* = 0.55) were not significant. Error bars indicate standard errors of the mean. CBP, patients with chronic lower back pain; CM, Patients with chronic migraine; HC, healthy controls.

Familiarity differed significantly between the three conditions (ME *condition*: *F*(2,340) = 8.81, *P* < 0.001, *η²_p_* = 0.05) but not between the three groups (ME *group*: *F*(2,170) = 2.23, *P* = 0.11, *η²_p_* = 0.03). There was no significant *group* × *condition* interaction (*F*(4,340) = 0.61, *P* = 0.65, *η²_p_* = 0.007; Fig. 3A; variability of the effects of pain on familiarity is shown in Supplementary Fig. 3A). According to post-hoc tests, familiarity was lower for *head pain* than for *no pain* (−0.16 ± 0.04, *t*(340) = −4.09, *P* < 0.001, *d* = 0.44) and for *back pain* than for *no pain* (−0.11 ± 0.04, *t*(340) = −2.87, *P* = 0.01, *d* = 0.31). However, familiarity did not differ between the two pain conditions (0.05 ± 0.04, *t*(340) = 1.22, *P* = 0.67, *d* = 0.13).

When calculating an LMM including only patients, neither the main effects (ME *group*: *F*(1,112) = 2.00, *P* = 0.16, *η²_p_* = 0.02; ME *condition*: *F*(1,112) = 0.79, *P* = 0.37, *η²_p_* = 0.007), nor the interaction of *group × condition* (*F*(1,112) = 0.08, *P* = 0.78, *η²_p_* = 0.001) were significant.

Similar to familiarity, *recollection* also differed significantly between the three conditions (ME *condition*: *F*(2,340) = 4.52, *P* = 0.01, *η²_p_* = 0.03; Fig. 3B; variability of the effects of pain on recollection is shown in Supplementary Fig. 3B). Post-hoc *t*-tests showed that recollection was significantly lower for *back pain* than for *no pain* (−0.04 ± 0.02, *t*(340) = −2.81, *P* = 0.02, *d* = 0.31), whereas recollection for *head pain* was not significantly different from *no pain* (−0.01 ± 0.02, *t*(340) = −0.49, *P* = 1.00, *d* = −0.05). The comparison of recollection in the *back pain* and *head pain* conditions did not reach significance (0.04 ± 0.02, *t*(340) = 2.33, *P* = 0.06, *d* = 0.25). Exploratory within-group comparisons of this trend-level effect showed that patients with chronic migraine exhibited significantly better recollection performance in the head pain condition compared to the back pain condition (*t*(340) = −2.242, *P* = 0.026). Again, no significant effects were observed for the factor *group* (ME *group*: *F*(2,170) = 1.03, *P* = 0.36, *η²_p_* = 0.01) and the *group* × *condition* interaction (*F*(4,340) = 0.76, *P* = 0.55, *η²_p_* = 0.01).

Comparing the two patient groups, the restricted LMM analysis revealed a significant main effect of *condition*, i.e. a stronger detrimental effect for *back pain* than *head pain* (*F*(1,112) = 9.08, *P* = 0.003, *η²_p_* = 0.03), but no ME of *group* (*F*(1,112) = 0.59, *P* = 0.44, *η²_p_* = 0.01) or *group × condition* interaction (*F*(1,112) = 0.39, *P* = 0.53, *η²_p_* = 0.01).

#### Modulation of the detrimental effects of pain

Covariate testing in both patient groups using model comparisons revealed that the effects of pain on *d’* and *familiarity* were both modulated by the participants’ score in the subscale ‘Fatigue and slowing down in practical activities’ (FEDA-EV) of the Questionnaire of Experienced Attention Deficits (FEDA).

**Figure 4:**
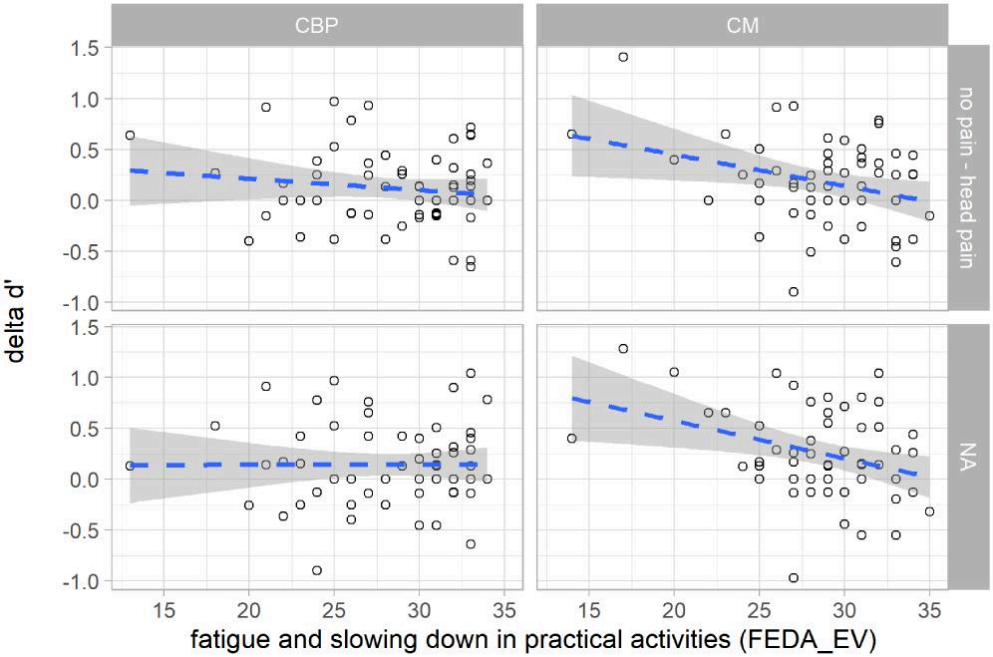
**Experienced attention deficits in everyday life modulate the effects of experimental pain on recognition performance in patients with migraine.** Circles represent individual data of patients with chronic migraine (CM, *n* = 55) and patients with chronic back pain (CBP, *n* = 59). Y-axis: d’ no pain—d’ back pain/head pain; positive values indicate impaired recognition performance for images paired with pain, negative values indicate better recognition performance for images paired with pain. Lower FEDA-EV scores (x-axis) indicate higher experienced attention deficits in everyday life. For visualization purposes, linear regression lines (dashed, blue) were fitted to the data using the method ‘lm’ from the R package ggpubr, separately for each combination of group and condition. Grey areas depict the 95% confidence intervals. FEDA_EV, Questionnaire of Experienced Attention Deficits, subscale ‘fatigue and slowing down in practical activities’.

For *d’,* the LMM including the covariate showed a significant *ME* of the *FEDA-EV score* (*F*(1,110) = 7.01, *P* = 0.009, *η²_p_* = 0.06) with the *group* × *FEDA-EV* interaction approaching significance (*F*(1,110) = 3.66, *P* = 0.06, *η²_p_* = 0.03). These effects were primarily driven by the CM group, in which the effect of pain on task performance (*d’*) was significantly modulated by the FEDA-EV score (ME *FEDA-EV*: *F*(1,53) = 8.57, *P* = 0.005, *η²_p_* = 0.14; Fig. 4). Given the strong association between *d’* and *familiarity,* results for the index familiarity were highly similar (ME *FEDA-EV: F*(1,110) = 6.01, *P* = 0.02, *η²_p_* = 0.05; *group × FEDA-EV* interaction: (*F*(1,110) = 3.61, *P* = 0.06, *η²_p_* = 0.03), indicating that patients with migraine who experience stronger attention deficits in everyday life also showed stronger disruptive effects of both head and back pain on recognition performance.

For *recollection,* fear of pain differentially modulated the effects of *back pain* and *head pain* in both patient groups, as indicated by significant interactions of *group × fear of pain* (*F*(1,215.49) = 6.33, *P* = 0.01, *η²_p_* = 0.03) and *condition × fear of pain* (*F*(1,117.40) = 4.18, *P* = 0.04, *η²_p_* = 0.03; for visualization, see Supplementary Fig. 4). Further analysis including only patients revealed that these effects were driven by the CBP group, where higher fear of pain ratings were related to reduced negative effects, regardless of the stimulation site (CBP group: *ME fear ratings*: *F*(1,109.38) = 5.93, *P* = 0.02, *η²_p_* = 0.02). In contrast, no significant effects of fear of pain were found in CM patients.

None of the other tested covariates (see *Methods* for a complete list) significantly modulated the outcomes of interest (*d’, familiarity, recollection*). Model comparison results are provided in Supplementary Table 7.

## Discussion

To our knowledge, this pre-registered study is the largest study (*n* = 173) to examine the effect of experimental pain on cognitive performance in patient groups with different types of chronic pain, namely chronic back pain and chronic migraine. The comparison with age-matched healthy participants revealed similar effects of short electrical pain stimuli on reaction times, categorization performance and, importantly, episodic memory (d’, familiarity, recollection) in healthy participants and both patient groups.

As reported previously,^25^ reaction times were faster when painful electrical stimuli were applied concurrently with images that required classification as living or non-living. Importantly, these effects did not differ between patients with chronic pain and healthy participants, suggesting that the attentional effects of pain are a general phenomenon, rather than one specific to chronic pain. We further confirmed that memory for images paired with painful stimuli was lower than for those presented without concurrent stimulation. Importantly, this negative effect of pain on memory performance was comparable across all three groups. No differences were found between the effect of experimental pain applied to the head or lower back and importantly, no interaction was detected between the type of chronic pain and the site of experimentally applied pain. Clinical and psychological parameters had little or no effect on the pain-induced impairment of recognition memory in patients with chronic pain.

As hypothesized and previously reported,^11,25,38,48^ experimentally applied pain disrupted the encoding of visual stimuli. Although both groups of patients were severely affected by chronic pain—almost 75% of patients had been in pain for more than 5 years—and patients reported stronger attention deficits in their daily lives than healthy participants, the impact of experimental pain on the various recognition measures was not significantly different from that in participants without chronic pain. This finding contrasts with previous reports of impaired long-term memory (in particular recollection) in chronic pain^24,49^ but aligns with studies reporting no effect of headache pain during encoding on recognition performance^50^ or even enhanced recollection-based memory.^51^

Although pain in the trigeminal system could be expected to have a greater disruptive effect due to its high biological relevance,^18,38^ our study did not find a heightened disruption by head pain. Even among migraine patients, who reported a greater fear of head pain than back pain stimuli, there was no observable increase in interference for head pain over back pain. Instead, the opposite pattern emerged: migraine patients showed better recollection-based memory for images previously paired with head pain than back pain on the descriptive level, suggesting that these images were remembered with greater confidence.^41^ This aligns with a recent study showing better recollection-based memory for negative and highly arousing visual stimuli in migraine patients than a healthy control group in an incidental encoding task.^51^ The authors concluded that migraineurs may be more sensitive to the valence and arousal of emotional stimuli. According to the dual-process model of memory,^41^ recollection-based memory involves retrieving both the item and the context in which it was encountered. One might speculate, that, in our study, those images previously presented with head pain were more arousing or significant, particularly for patients with migraine, leading to better recognition. Future studies should incorporate additional behavioural measures (e.g. source memory tasks) and psychophysiological or neuroimaging methods to explore the mechanisms underlying potentially pain-related facilitating effects on recollection.

Although recognition memory disruption from experimental pain was similar across groups, our extended neuropsychological battery identified distinct cognitive differences: migraine patients showed reduced verbal flexibility, while chronic back pain patients demonstrated poorer divided attention compared to healthy controls. These findings suggest that chronic pain may not uniformly affect all cognitive domains but instead preferentially impacts executive and attentional control systems. Thus, the lack of group differences in pain-induced recognition memory should not be interpreted as an absence of cognitive consequences of chronic pain; rather, it points to differential vulnerability of cognitive domains, with executive and attentional processes more affected than episodic memory encoding in acute laboratory conditions.

A recent reanalysis of similar studies conducted in our lab (*n* = 247 healthy participants) found no relevant modulation of the detrimental effect of pain on recognition memory by expectation, pain catastrophizing and pain-related fear.^52^ This large study, involving patients with chronic back pain or migraine, similarly shows that the disruptive effects of experimental pain on memory are largely unaffected by pain-related cognitions or clinical parameters (such as clinical pain intensity or pain frequency). Notably, only experienced attention deficits in daily life (in migraine patients) and fear of pain (in back pain patients) appear to modify the effects of experimental pain on recognition memory.

Expectation is typically a key factor influencing pain perception and its interaction with cognition. In this study, however, expectation of pain-task interference did not influence actual task interference. It should be noted that expectations were not separately assessed for each pain condition, and participants were unaware of the subsequent recognition task when providing their ratings. As such, these ratings most likely referred to the categorization task. While a recent study in healthy participants found no differences in expected interruptive effects between visceral and thermal cutaneous pain,^48^ it remains plausible that expectations regarding the effects of pain applied to the head and back vary, especially between the patient groups. Future studies should consider capturing condition-specific expectations to clarify their potential role in pain-cognition interactions.

The divergent reports on the influence of clinical or cognitive factors on the disruptive effects of pain in chronic pain patients,^24,53-56^ along with our findings, highlight significant variability in the susceptibility of pain patients to pain-induced disruption of higher-order cognitive processes. The extent of pain-cognition interference likely depends on a complex interplay of various stimulus-related factors, an individuals’ psychological traits and states as well as clinical variables. Future research with larger sample sizes is needed to clarify and disentangle these contributing factors.

### Limitations

Although the brief electrical stimuli reliably reduced recognition performance, the controlled and highly artificial experimental design, along with the type of pain stimulation used, limits the generalizability of the results to the effects of clinical pain. The brief electrical stimuli were applied in an environment where participants had the option to withdraw from the study at any time, which made the painful experience highly controllable and potentially less threatening—particularly compared to the unpredictability of chronic pain. Variations of our design that account for these differences may be able to explore the influence of such variables in more detail. While this study was not designed to test the specificity of pain-related interference, the exclusive use of painful stimuli limits conclusions about whether the effects are unique to pain. Another limitation is the potential influence of unmeasured confounders, such as psychological or contextual factors. Although common in group comparison studies (e.g. with and without chronic pain), this should be considered when interpreting group differences.

This study examined pain's effect on episodic memory during encoding. However, pain may impact other memory types differently. Additionally, the timing of pain application is likely critical (e.g. during encoding, consolidation or retrieval). Furthermore, it remains an open question whether pain-related cognitive impairments generalize across domains, or if certain domains are more or less vulnerable. Advancing our understanding of pain-cognition interactions requires systematic investigation of these domain- and phase-specific effects in both healthy populations and individuals with chronic pain.

## Conclusion and outlook

This study, conducted in *n* = 173 participants, provides the first large-scale exploration of the interruptive effect of pain and the factors that influence it in a large sample of chronic pain patients. Notably, the findings present a largely ‘positive picture’ for the studied pain disorders. Neither patient groups showed a generally heightened susceptibility to the disruptive effects of experimental pain or an impairment in general cognitive functioning, despite the presence of maladaptive pain-related cognitions and psychiatric symptoms.

The marked inter-individual variability in pain-induced memory impairments highlights the need for more nuanced approaches beyond group-level analyses. Larger and more diverse samples could enable person-centred methods to investigate how cognitive, clinical, and situational factors interact in shaping performance under pain, as well as how long-term neuroplastic or neurodegenerative changes linked to chronic pain impact this pain-cognition interaction. Additionally, repeated-measures designs may help capture the dynamic nature of these effects over time and identify factors driving within-person variability. Such approaches are essential to better understand individual differences in pain-cognition interactions. Only through a comprehensive understanding of the interruptive effect of pain and its modulating factors can we develop more effective therapeutic interventions for patients who are impaired in their daily lives by cognitive impairments related to acute and, particularly, chronic pain syndromes.

## Supplementary Material

fcaf486_Supplementary_Data

## Data Availability

The data and analysis code that support the findings of this study are openly available in the Open Science Framework (OSF) at https://osf.io/sk26g/.
