# Supplementary material for: The impact of pain on memory: a study in chronic low back pain and migraine patients
Source: Brain Commun. 2025 Dec 10;8(1):fcaf486. doi: 10.1093/braincomms/fcaf486 (PMC12776015; doi:10.1093/braincomms/fcaf486)
Supplement: fcaf486_Supplementary_Data [file fcaf486_supplementary_data.docx]

# **Supplementary Information:**

**The Impact of Pain on Memory: A Study in Chronic Low Back Pain and Migraine Patients**

Katarina Forkmann^1^, Vanessa C. Dobischat^1^, Katharina Schmidt^1^, Katrin Scharmach^1^, Dagny Holle^3^, Katja Wiech^1, 2^, Ulrike Bingel^1^

Author affiliations:

^1^ Clinic for Neurology, Center for Translational Neuro- and Behavioral Sciences (C-TNBS), University Hospital Essen, University of Duisburg-Essen, Essen, Germany

^2^ Wellcome Centre for Integrative Neuroimaging (WIN), Nuffield Department of Clinical Neurosciences, University of Oxford, John Radcliffe Hospital, Oxford, UK

^3^ Clinic for Neurology, Centre for Translational Neuro- and Behavioral Sciences (C-TNBS), West German Headache Centre, University Hospital Essen, University of Duisburg-Essen, Essen, Germany.

Correspondence to:

Katarina Forkmann
Clinic for Neurology
University Hospital Essen
Hufelandstr. 55
45147 Essen
Germany

[Katarina.forkmann@uk-essen.de](mailto:Katarina.forkmann@uk-essen.de)

## **Supplementary Analyses**

**Changes in stimulus intensity applied during the categorization task**

Since repetitive electrical stimulation frequently leads to habituation, which would result in decreasing pain perception throughout the experiment, stimulus intensities were manually adjusted throughout the categorization task depending on the participants’ pain intensity ratings. Using a linear mixed model approach, we tested, whether stimulus intensities (mA) differed between conditions, groups and time (start of the categorization task vs. end of the task) as well as for any significant interaction. This analysis showed significant main effects of *condition* (*F*(1,510) = 7.305, *p* = 0.007) and *time* (*F*(1,510) = 168.10, *p* < 0.001) while no other effect (main effect of *group* or interaction) yielded significant results (all *p* > 0.55), indicating that stimulus intensities were higher for the *back pain* condition than the *head pain* condition and that stimulus intensities were significantly higher at the end of the experiment compared to the beginning, reflecting habituation (see figure below). Importantly, stimulus intensities increased similarly over time for each group and each condition and overall, groups did not differ with respect to applied stimulus intensities.


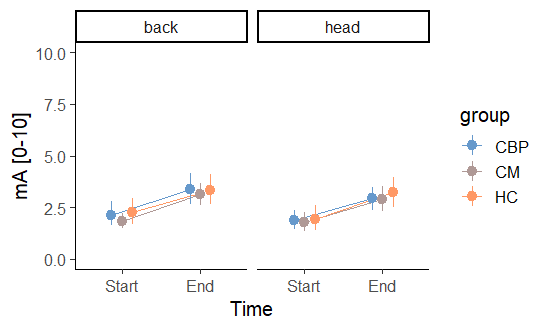


CBP, patients with chronic lower back pain; CM, Patients with chronic migraine; HC, healthy controls; mA, milli ampere.

**Changes in pain intensity ratings throughout the categorization task**

To counteract habituation effects to repetitive electrical stimulation and to ensure sufficiently high pain intensity ratings throughout the task, stimulus intensities were manually adjusted (see above). In an exploratory analysis, we analyzed corresponding changes in pain intensity ratings using a linear mixed model including the factors *trial number* (1 - 20), *group*, and *condition*. We found a significant main effect of *trial number* (*F*(1,170) = 51.942, *p* < 0.001), indicating a significant change of pain intensity ratings over time. In addition the *group x condition* interaction (*F*(2,6568) = 8.696, *p* < 0.001) and the interaction of *group x condition x trial number* (*F*(2,6568) = 6.074, *p* = 0.002) were significant. Importantly, post-hoc comparisons (Bonferroni-corrected) showed, that pain intensity ratings developed differently between *head* *pain* and *back pain* condition for the HC group only (*t*(6568) = 3.184, *p* = 0.022). All other pairwise comparisons did not reach significance. Together, this analysis indicates that pain intensity ratings increased over time (due to the manual adjustments of stimulus intensities) and that the adjustment of stimulus intensities led to sufficiently high pain perception throughout the task. Importantly, changes in pain intensity ratings were comparable between groups and conditions (see figure below).


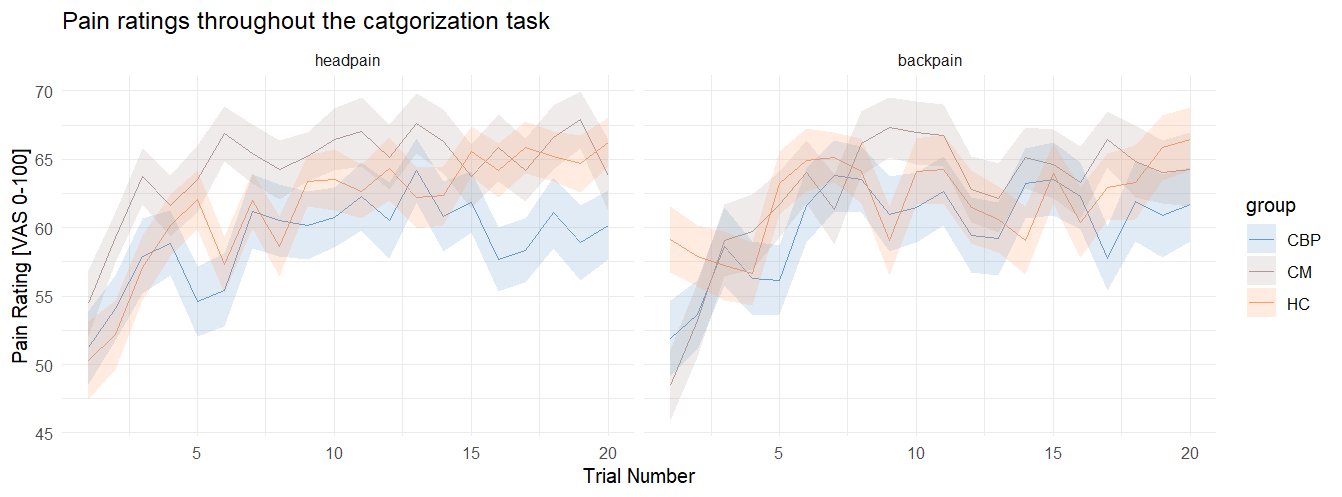


CBP, patients with chronic lower back pain; CM, Patients with chronic migraine; HC, healthy controls; VAS, visual analogue scale.

## **Supplementary Figures**

**A**


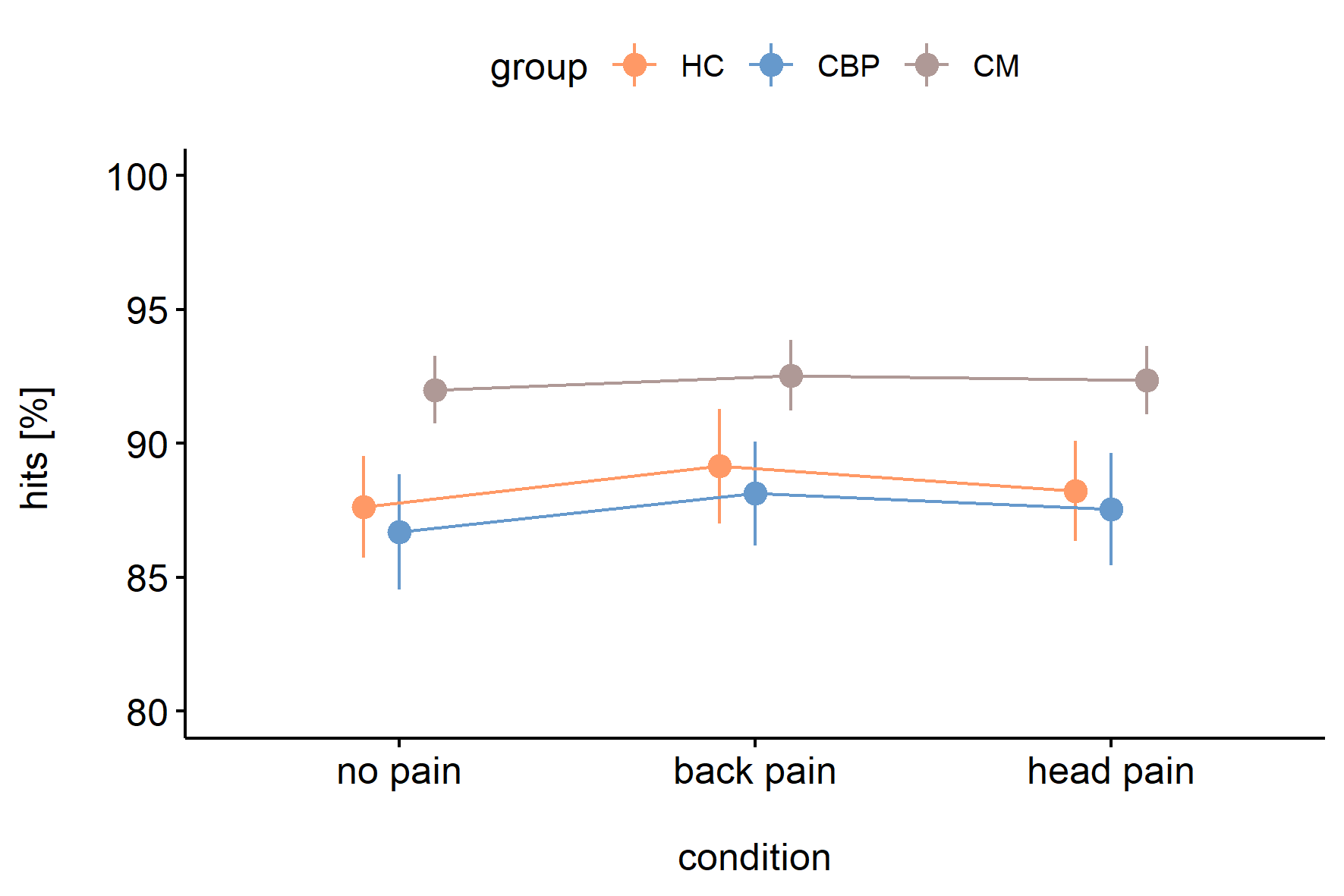


**B**


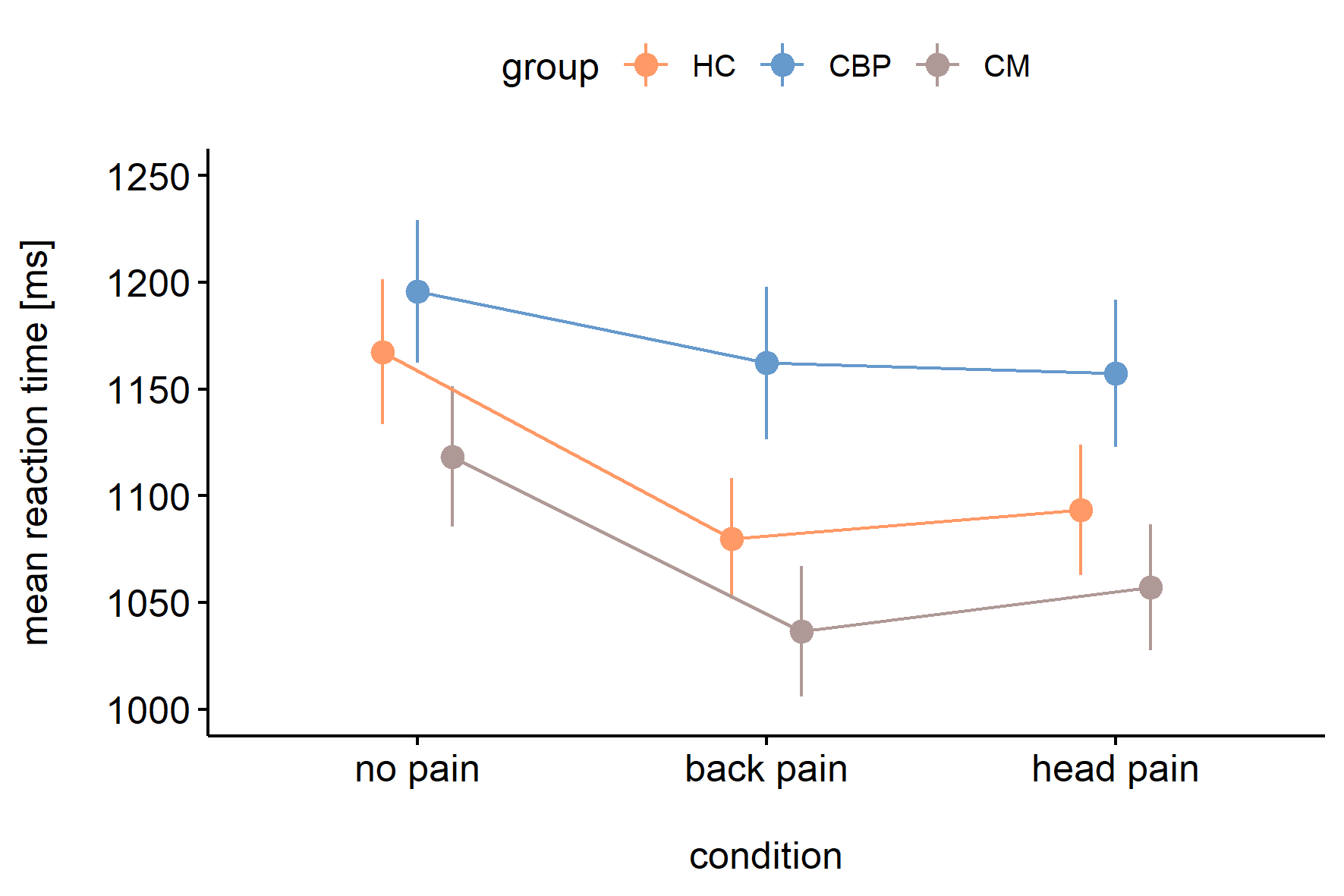


**Supplementary Figure 1 Painful stimulation reduces reaction times but does not affect categorization accuracy during a categorization task.** Mean values for categorization accuracy (%) **(A)** and mean reaction times (ms) **(B)** are shown across the three experimental conditions (no pain, back pain, head pain) and for each group (HC, n = 59; CM, n = 55; CBP, n = 59). Error bars indicate standard error of the mean. Data were statistically analyzed using linear mixed models. For *hits*, analyses revealed no significant effects (main effect *condition*: *F*(2,340) = 1.70, *p* = 0.18; main effect *group*: *F*(2,170) = 2.21, *p* = 0.11; *group x condition*: *F*(4,340) = 0.13, *p* = 0.97). For *reaction times*, analyses showed a significant main effect of *condition* (*F*(2,340) = 27.75, *p* < 0.001; post hoc testing: no pain vs head pain, *t*(340) = 5.91, *p* < 0.001; no pain vs back pain, *t*(340) = 6.89, *p* < 0.001; head pain vs back pain, *t*(340) = 0.98, *p* = 0.98), but no significant main effect of *group* (*F*(2,170) = 2.69, *p* = 0.07) or *group x condition* interaction (*F*(4,340) = 1.61, *p* = 0.17). CBP, patients with chronic lower back pain; CM, Patients with chronic migraine; HC, healthy controls; ms, milliseconds.


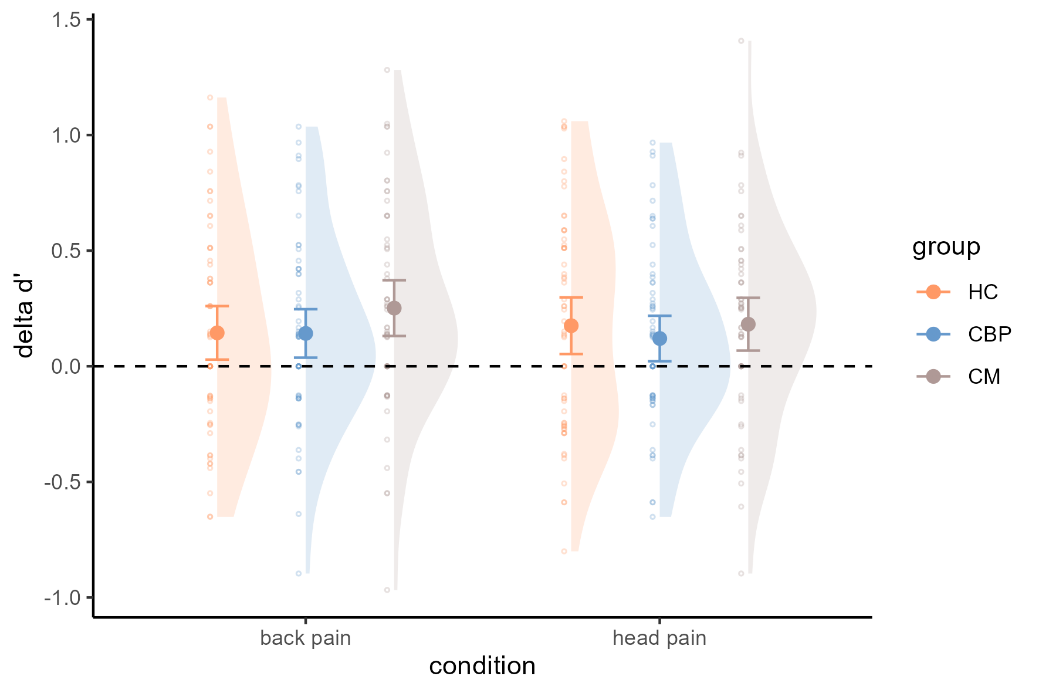


**Supplementary Figure 2 Variability of the effects of pain on recognition memory (d’)**. The graph illustrates the effect of pain on recognition performance (delta d’). Each dot represents individual subject data for the back pain condition (i.e., d’ no pain – d’ back pain) and the head pain condition (d’ no pain – d’ head pain). Shown are the mean values, 95% confidence interval (error bar) and the data distribution for the two pain conditions across the three groups (HC, n = 59; CBP, n = 59; CM, n = 55). Positive values indicate lower recognition performance for images previously paired with pain. Negative values reflect improved recognition performance for such images. CBP, patients with chronic lower back pain; CM, Patients with chronic migraine; HC, healthy controls.


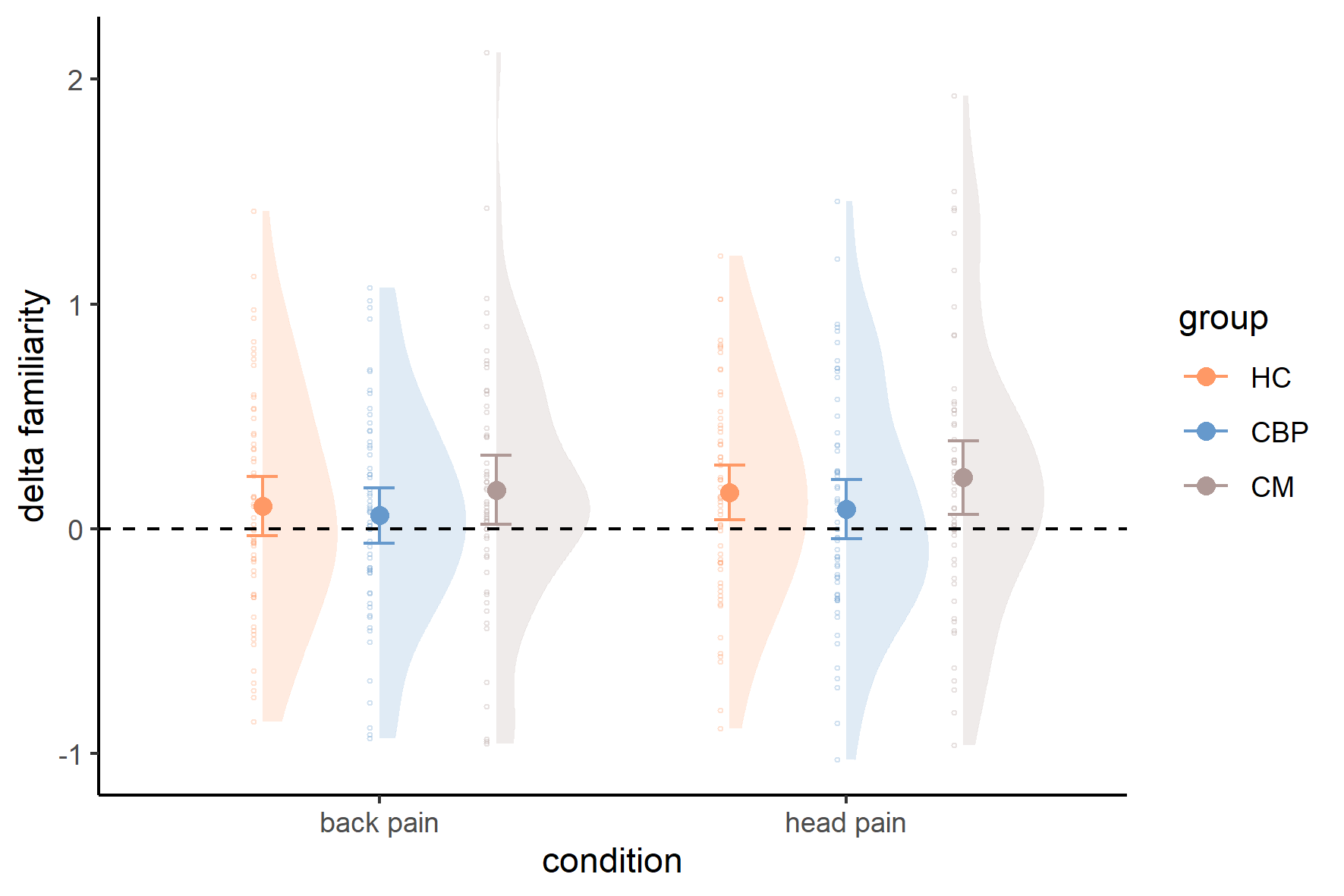


**A**


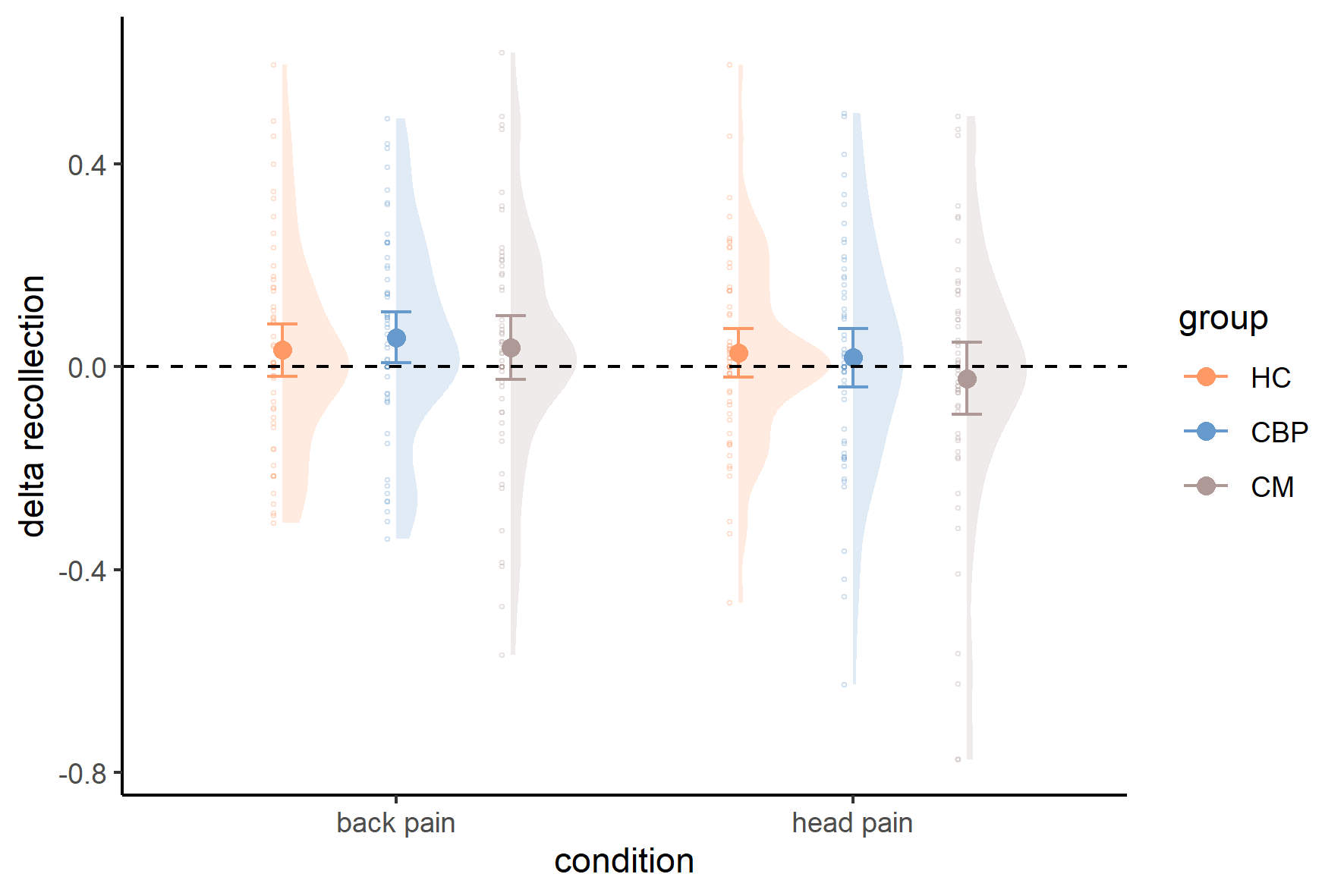


**B**

**Supplementary Figure 3 Variability of the effects of pain on familiarity-based and recollection-based memory**. The graph illustrates the effect of pain on familiarity-based memory (delta familiarity, **A**) and recollection-based memory (delta recollection, **B**). Each dot represents individual subject data for the back pain condition (i.e., familiarity no pain – familiarity back pain) and the head pain condition (i.e., familiarity no pain – familiarity head pain). Shown are the mean values, 95% confidence interval (error bar) and the data distribution of the data for the two pain conditions and the three groups (HC, n = 59; CBP, n = 59; CM, n = 55). Positive values indicate impaired familiarity-based or recollection-based memory for images previously paired with pain. Negative values indicate improved familiarity-based or recollection-based memory for such images. CBP, patients with chronic lower back pain; CM, Patients with chronic migraine; HC, healthy controls.


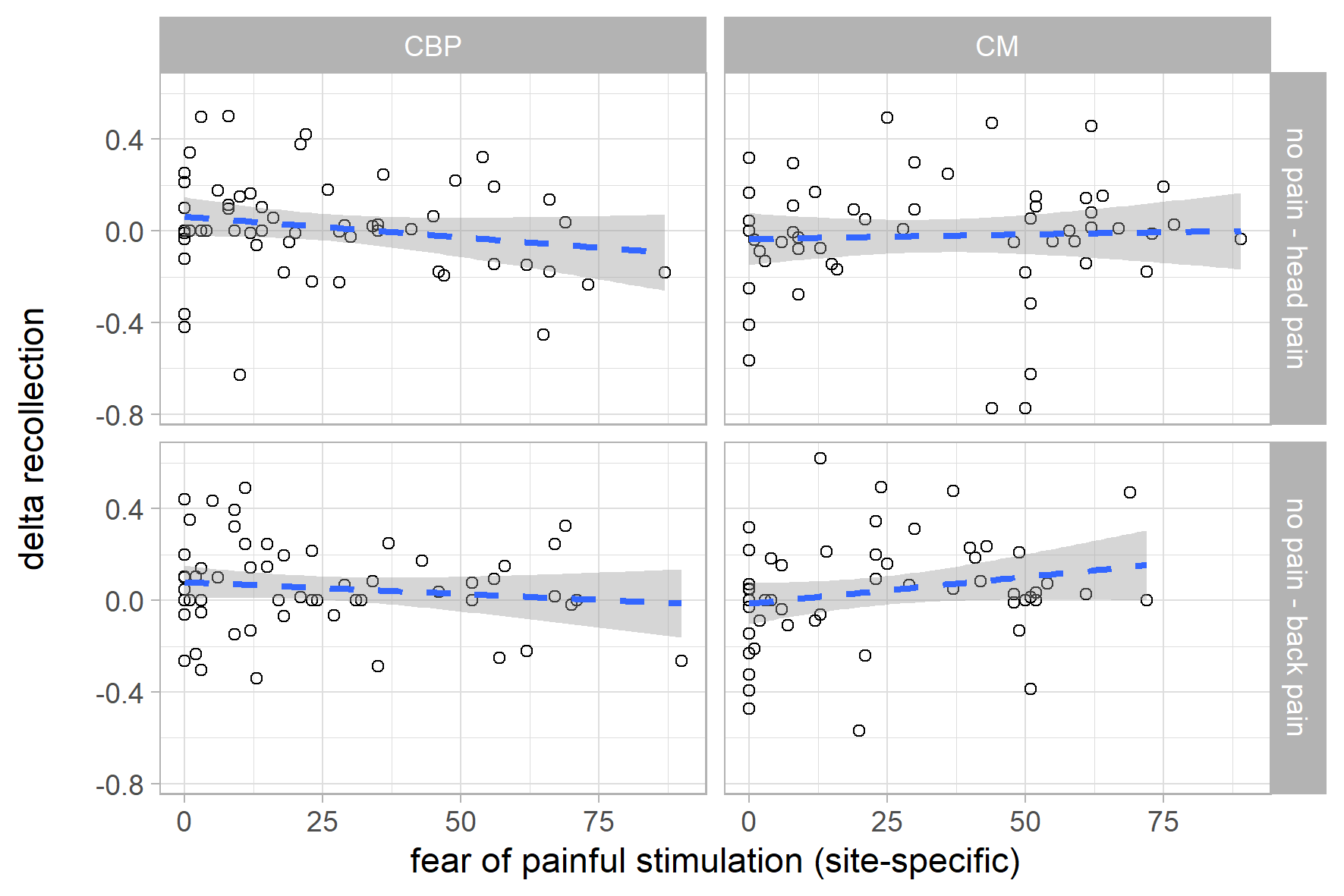


**Supplementary Figure 4 Fear of painful stimulation differentially influences the effect of pain on recollection-based memory in patients with chronic back pain (n = 59) and patients with chronic migraine (n = 55).** Circles represent individual participant data (y-axis: delta recollection = no pain – head pain (top); no pain – head pain (bottom). Positive values indicate impaired recognition performance for images paired with pain, negative values indicate better recognition performance for images paired with pain. Ratings for fear of painful stimulation (x-axis) could range from 0 – 100. For visualization purposes, linear regression lines (dashed, blue) were fitted to the data using the method “lm” from the R package ggpubr, separately for each combination of group and condition. Grey areas depict the 95% confidence intervals. CBP, patients with chronic lower back pain; CM, Patients with chronic migraine.

## **Supplementary Tables**

**Supplementary Table 1 Sample description: Pain presence, intensity, and analgesic use across study days (descriptives and group comparisons)**

|  | **Group** | | | **Inference** |
| --- | --- | --- | --- | --- |
| **Variable** | HC | CBP | CM |  |
| N | 59 | 59 | 55 |  |
| **Currently in pain (study day 1)** | | | | |
| Yes | 1 (1.7 %) | 48 (81.4 %) | 33 (60.0 %) | *χ²(1) = 5.32,  p = 0.02*^§^* |
| No | 57 (96.6 %) | 11 (18.6 %) | 21 (38.2 %) |  |
| NA | 1 (1.7 %) | - | 1 (1.8 %) |  |
| **Currently in pain (study day 2)** | | | | |
| Yes | 5 (8.5 %) | 48 (81.4 %) | 30 (54.5 %) | *χ²(1) = 8.27,  p = 0.004*^§^* |
| No | 54 (91.5 %) | 11 (18.6 %) | 23 (41.8 %) |  |
| NA | - | - | 2 (3.6 %) |  |
| Pain intensity on study day 1 [VAS 0-100]^#^ | 12 ± 0 | 28.46 ± 19.24 | 34.61 ± 22.61 | *W* = 662,  *p* = 0.21^§^ |
| Pain intensity on study day 2  [VAS 0-100]^#^ | 7.4 ± 4.62 | 27.62 ± 22.34 | 28.63 ± 21.53 | *W* = 668.5,  *p* = 0.60^§^ |
| **Pain medication within 24 hours before study day 1 (neuropsychological testing)** | | | | |
| Yes |  | 10 (16.9 %) | 9 (16.4 %) | *χ²*(1) < 0.001, *p* = 1^§^ |
| No |  | 49 (89.1 %) | 43 (78.2 %) |  |
| NA |  | - | 3 (5.5 %) |  |
| **Pain medication within 24 hours before study day 2 (encoding & recognition)** | | | | |
| Yes |  | 12 (20.3 %) | 13 (23.6 %) | *χ*²(1) = 0.04,  *p* = 0.84^§^ |
| No |  | 47 (79.7 %) | 41 (74.5 %) |  |
| NA |  | - | 1 (1.8 %) |  |

^#^ Descriptive data are reported only for those study participants who reported pain on study day 1 or 2.
**P* < 0.05. ^§^ Statistical comparison of CM and CBP group.
CBP, patients with chronic lower back pain; CM, Patients with chronic migraine; HC, healthy controls; NA, not available; VAS, visual analogue scale.

**Supplementary Table 2 Neuropsychological test procedures and subtests used**

| **Neuropsychological test procedure** | **Subtest** |
| --- | --- |
| Verbal learning and memory test (VLMT)^1^ | Learning; interference; recognition |
| Wechsler-Memory-Scale Revised (WMS-R)^2^ | Digit span forward; Digit span backward |
| Trail Making Test (TMT)^3^ | TMT-A; TMT-B |
| Regensburger Word Fluency Test (RWT)^4^ | Letter: S; Alternating letters: G/R; Caterogy: animals; Alternating categories: sports/fruits |
| Tests for Attentional Performance (TAP)^5^ | Divided attention; interference; cognitive flexibility |

**Supplementary Table 3 Questionnaire data. Descriptive data (M ± SD) and inference.**

|  | **Group** | | | | **Inference** | | **Post-hoc tests** | | | |
| --- | --- | --- | --- | --- | --- | --- | --- | --- | --- | --- |
| **Variable** | HC | CBP | CM |  | | HC - CBP | | HC - CM | CBP – CM |  |
| DASS-A | 1.08 ± 1.76 | 2.91 ± 3.05  (n = 56) | 4.51 ± 3.69  (n = 53) | *Χ²(2) =39.34,  p < 0.001** | | *W = 2369, p < 0.001** | | *W = 2566, p < 0.001** | *W = 1070, p = 0.034** |  |
| DASS-S | 3.34 ± 3.29 | 6.39 ± 4.16  (n = 56) | 8.96 ± 5.07  (n = 53) | *Χ²(2) =39.28,  p < 0.001** | | *W = 2389, p < 0.001** | | *W = 2573, p < 0.001** | *W = 1056, p = 0.028** |  |
| DASS-D | 1.42 ± 1.69 | 4.54 ± 4.65  (n = 56) | 5.40 ± 4.43  (n = 53) | *Χ²(2) =31.21,  p < 0.001** | | *W = 2337, p < 0.001** | | *W = 2484, p < 0.001** | W = 1260, p = 0.516 |  |
| PASS total | 23.58 ± 12.81 | 31.64 ± 16.58 | 42.56 ± 13.48  (n = 54) | *Χ²(2) =41.35,  p < 0.001** | | *W = 2220, p = 0.03** | | *W = 2748, p < 0.001** | *W = 942,  p = 0.003** |  |
| PCS | 9.14 ± 8.16 | 18.90 ± 10.59 | 21.25 ± 9.16 | *Χ²(2) =41.51,  p < 0.001** | | *W = 2634, p < 0.001** | | *W = 2693, p < 0.001** | W = 1382, p = 0.516 |  |
| STAI_trait | 33.32 ± 9.02 | 41.98 ± 10.77  (n = 57) | 42.83 ± 10.54  (n = 54) | *Χ²(2) =27.84,  p < 0.001** | | *W = 2473, p < 0.001** | | *W = 2404, p < 0.001** | W = 1436, p = 1 |  |
| FEDA- EV^#^ | 32.42 ± 2.37 | 28.25 ± 4.60 | 28.73 ± 4.22 | *Χ²(2) =41.68,  p < 0.001** | | *W = 687,  p < 0.001** | | *W = 672,*  *p < 0.001** | W = 1550, p = 1 |  |
| FEDA-AV^#^ | 51.63 ± 4.14 | 47.86 ± 7.04 | 45.35 ± 7.94 | *Χ²(2) =20.98,  p < 0.001** | | *W = 1185, p = 0.008** | | *W = 840,*  *p < 0.001** | W = 1914, p = 0.296 |  |
| FEDA-AM^#^ | 25.68 ± 3.25 | 22.27 ± 4.93 | 21.00 ± 5.07 | *Χ²(2) =29.48,*  *p < 0.001** | | *W = 994,  p < 0.001** | | *W = 736,*  *p < 0.001** | W = 1899, p = 0.351 |  |
| HIT-6 | - | - | 62.96 ± 4.37  (n = 54) | - | | - | | - | - |  |

^#^ lower values correspond to higher perceived attention impairments. **P* < 0.05.

CBP, patients with chronic low back pain; CM, patients with chronic migraine; DASS-A, Depression Anxiety Stress Scale – Subscale Anxiety; DASS-D, Depression Anxiety Stress Scale – Subscale Depression; DASS-S, Depression Anxiety Stress Scale – Subscale Stress; FEDA-AM, Questionnaire of Experienced Attention Deficits - subscale reduction in motivation; FEDA-AV, Questionnaire of Experienced Attention Deficits - subscale distractibility and slowing down in mental processes; FEDA-EV, Questionnaire of Experienced Attention Deficits - subscale fatigue and slowing down in practical activities; HC, Healthy controls; HIT-6, Headache-Impact-Test; PASS total, Pain Anxiety Symptom Scale – sum score; PCS, Pain Catastrophizing Scale; STAI trait, State Trait Anxiety Inventory - trait scale.

**Supplementary Table 4 Neuropsychological data. Descriptive data (M ± SD) and inference.**

|  | **Group** | | | | **Inference** | | **Post-hoc tests** | | | |
| --- | --- | --- | --- | --- | --- | --- | --- | --- | --- | --- |
| **Variable** | HC | CBP | CM |  | | HC – CBP | | HC - CM | CBP - CM |  |
| **VLMT** |  |  |  |  | |  | |  |  |  |
| VLMT 1-5 | 71.44 ± 26.05 | 69.75 ± 26.05 | 68.18 ± 24.12 | χ²(2) =1.20,  p = 0.55 | | - | | - | - |  |
| VLMT 5-6 | 55.68 ± 29.96 | 54.92 ± 30.67 | 57.27 ± 26.7 | χ²(2) =0.167,  p = 0.92 | | - | | - | - |  |
| VLMT 5-7 | 57.27 ± 28.92 | 56.78 ± 32.94 | 58.82 ± 28.63 | χ²(2) =0.004,  p = 1.00 | | - | | - | - |  |
| VLMT recognition | 74.20 ± 29.29 | 73.14 ± 30.44 | 80.55 ± 24.81 | χ²(2) =1.575,  p = 0.46 | | - | | - | - |  |
| VLMT recognition errors | 64.85 ± 32.32 | 57.03 ± 33.31 | 65.27 ± 33.57 | χ²(2) =2.335,  p = 0.31 | | - | | - | - |  |
| **WMS** |  |  |  |  | |  | |  |  |  |
| WMS forward | 55.93 ± 29.12 | 60.29 ± 27.53 | 50.82 ± 32.5 | χ²(2) =2.015,  p = 0.37 | | - | | - | - |  |
| WMS backwards | 65.14 ± 25.99  (n = 58) | 59.73 ± 27.55 | 60.18 ± 29.32 | χ²(2) =1.043,  p = 0.59 | | - | | - | - |  |
| **TMT** |  |  |  |  | |  | |  |  |  |
| TMT-A | 62.98 ± 25.49  (n = 57) | 58.98 ± 28.27 | 51.30 ± 28.02  (n = 54) | χ²(2) =5.185,  p = 0.07 | | - | | - | - |  |
| TMT-B | 64.29 ± 22.31  (n = 56) | 59.66 ± 27.40  (n = 58) | 56.11 ± 26.09  (n = 54) | χ²(2) =2.436,  p = 0.30 | | - | | - | - |  |
| **RWT** |  |  |  |  | |  | |  |  |  |
| RWT S | 59.63 ± 28.58 | 54.05 ± 29.25 | 47.42 ± 25.48 | χ²(2) =5.475,  p = 0.06 | | - | | - | - |  |
| RWT G-R | 48.66 ± 30.70 | 42.12 ± 31.75 | 35.25 ± 28.79 | χ²(2) =5.218,  p = 0.07 | | - | | - | - |  |
| RWT animals | 48.69 ± 27.73 | 50.93 ± 32.68 | 44.64 ± 27.76 | χ²(2) =1.271,  p = 0.53 | | - | | - | - |  |
| RWT fruits-sports | 63.36 ± 27.99 | 63.93 ± 27.55 | 50.96 ± 27.43 | *χ²(2) =8.508,*  *p = 0.01** | | p = 1.00 | | *p = 0.03** | *p = 0.03** |  |
| **TAP** |  |  |  |  | |  | |  |  |  |
| Divided attention (number of missed signals) | 50.90 ± 7.22 | 46.49 ± 7.85 | 47.70 ± 9.21 | *χ²(2) =9.372,  p = 0.009** | | *p = 0.006** | | p = 0.20 | p = 1.00 |  |
| Incompatibility (incompatibility index)^a^ | 53.53 ± 10.36 | 51.88 ± 8.62 | 52.67 ± 8.82 | χ²(2) =0.906,  p = 0.64 | | - | | - | - |  |
| Flexibility (overall performance) | 50.43 ± 8.41 | 47.03 ± 9.52 | 46.94 ± 10.11 | χ²(2) =3.638,  p = 0.16 | | - | | - | - |  |
| Flexibility (speed-accuracy-tradeoff)^b^ | 55.02 ± 9.91 | 55.51 ± 9.16 | 56.54 ± 7.39 | χ²(2) =0.454,  p = 0.80 | | - | | - | - |  |

Mean and Standard deviation of the percentile ranks (age, gender and education adjusted, where available) for all tests (except the TAP). Values for the TAP depict T-values (range: 20-80). ^a^ lower T-values correspond to stronger incompatibility effects. ^b^ lower T-values indicate a speed strategy (relatively high error rate for short response times), high T-values indicate an accuracy strategy (relatively low error rate for long response times). **P* < 0.05.

CBP, patients with chronic low back pain; CM, patients with chronic migraine; HC, Healthy controls; RWT, Regensburger word fluency test; TAP, Tests for Attentional Performance; TMT, Trail Making Test; VLMT, Verbal learning and memory test; WMS-R, Wechsler-Memory-Scale Revised

**Supplementary Table 5 Correlations between expected pain-task interference and disease parameters, psychological and pain-related variables**

|  | **Expectation of pain-task interference**^1^ | | | | | | | | | |  |
| --- | --- | --- | --- | --- | --- | --- | --- | --- | --- | --- | --- |
|  | HC | | | CM | | | | CBP | | |  |
| **Variable** | *r* | | *p* | | *r* | | *p* | | *r* | | *p* |
| DASS-A | -0.16 | | 0.22 | | 0.09 | | 0.53 | | 0.01 | | 0.95 |
| DASS-S | 0.00 | | 0.98 | | -0.14 | | 0.32 | | 0.08 | | 0.56 |
| DASS-D | -0.24 | | 0.06 | | -0.15 | | 0.28 | | 0.08 | | 0.57 |
| PASS total | -0.08 | | 0.53 | | 0.00 | | 0.98 | | 0.13 | | 0.31 |
| PCS | -0.06 | | 0.66 | | -0.11 | | 0.41 | | 0.20 | | 0.14 |
| STAI_trait | -0.22 | | 0.09 | | -0.18 | | 0.19 | | -0.04 | | 0.80 |
| FEDA-EV^2^ | 0.19 | | 0.15 | | -0.04 | | 0.78 | | -0.03 | | 0.80 |
| FEDA-AV^2^ | 0.11 | | 0.41 | | 0.01 | | 0.93 | | -0.20 | | 0.14 |
| FEDA-AM^2^ | 0.22 | | 0.09 | | 0.07 | | 0.63 | | -0.13 | | 0.33 |
| Pain days | - | | - | | 0.04 | | 0.80 | | -0.10 | | 0.45 |
| Current clinical pain intensity (GPQ) | - | | - | | -0.01 | | 0.96 | | -0.05 | | 0.69 |
| Average clinical pain intensity (GPQ) | - | | - | | 0.03 | | 0.84 | | 0.17 | | 0.21 |
| Disease severity (von Korff grade GPQ) | - | | - | | 0.10 | | 0.48 | | -0.01 | | 0.93 |
| HIT-6 | - | | - | | -0.04 | | 0.76 | | - | | - |
|  |  |  | |  | |  | |  | |  |  |

^1^ For easier interpretation of correlation coefficients, expectation ratings were inverted (higher values now indicate higher expectation of pain-task interference). ^2^ FEDA: lower values correspond to higher perceived attention impairments.

CBP, patients with chronic low back pain; CM, patients with chronic migraine; DASS-A, Depression Anxiety Stress Scale – Subscale Anxiety; DASS-S, Depression Anxiety Stress Scale – Subscale Stress; DASS-D, Depression Anxiety Stress Scale – Subscale Depression; FEDA-EV, Questionnaire of Experienced Attention Deficits - subscale fatigue and slowing down in practical activities; FEDA-AV, Questionnaire of Experienced Attention Deficits - subscale distractibility and slowing down in mental processes; FEDA-AM, Questionnaire of Experienced Attention Deficits - subscale reduction in motivation; GPQ, German Pain Questionnaire; HC, Healthy controls; HIT-6, Headache-Impact-Test; PASS total, Pain Anxiety Symptom Scale – sum score; PCS, Pain Catastrophizing Scale; STAI trait, State Trait Anxiety Inventory - trait scale.

**Supplementary Table 6 Statistical inference for pain thresholds, stimulus intensities of calibrated electrical stimuli and pain intensity ratings during the categorization task.**

|  | *df* | *F* | *p* |
| --- | --- | --- | --- |
| Pain threshold | | | |
| Condition | *1,169* | *20.316* | *0.001** |
| Group | 2,169 | 0.067 | 0.95 |
| group x condition | 2,169 | 0.067 | 0.94 |
| Calibrated electrical stimulus intensity | | | |
| Condition | *1,170* | *4.705* | *0.03** |
| Group | 2,170 | 0.609 | 0.54 |
| group x condition | 2,170 | 0.538 | 0.58 |
| Pain intensity rating | | | |
| Condition | 1,170 | 0.019 | 0.89 |
| Group | 2,170 | 2.668 | 0.07 |
| group x condition | 2,170 | 1.210 | 0.30 |

**P* < 0.05.

**Supplementary Table 7 Model comparison of Linear Mixed Models with and without potential covariates of interest.**

|  | ***AIC*** | ***χ²*** | ***df*** | ***p*** |
| --- | --- | --- | --- | --- |
| **d’ (no pain – pain)** |  |  |  |  |
| (condition * group) vs.  (condition * group * fear) | 215.50  222.74 | 0.752 | 4 | 0.94 |
| (condition * group) vs.  (condition * group * expected interruption) | 217.47  218.55 | 6.927 | 4 | 0.14 |
| (condition * group) vs.  (condition * group * PCS) | 218.19  222.80 | 3.390 | 4 | 0.49 |
| (condition * group) vs.  (condition * group * FEDA_EV) | *218.19*  *215.45* | *10.738* | *4* | *0.03** |
| (condition * group) vs.  (condition * group * FEDA_AV) | 218.19  221.01 | 5.178 | 4 | 0.27 |
| (condition * group) vs.  (condition * group * FEDA_AM) | 218.19  224.26 | 1.931 | 4 | 0.75 |
| (condition * group) vs.  (condition * group * HIT-6) ^a^ | 112.43  114.97 | 1.455 | 2 | 0.48 |
| (condition * group) vs. (condition * group * pain days) | 215.97  222.08 | 1.891 | 4 | 0.76 |
| (condition * group) vs.  (condition * group * current clinical pain intensity) | 216.92  219.24 | 5.686 | 4 | 0.22 |
| (condition * group) vs.  (condition * group * average clinical pain intensity) | 214.57  220.17 | 2.402 | 4 | 0.66 |
| (condition * group) vs.  (condition * group * von Korff grade) | 214.57  220.52 | 2.053 | 4 | 0.73 |
|  |  |  |  |  |
| **Familiarity (no pain – pain)** |  |  |  |  |
| (condition * group) vs.  (condition * group * fear) | 325.50  329.32 | 4.176 | 4 | 0.38 |
| (condition * group) vs.  (condition * group * expected interruption) | 326.50  329.23 | 5.273 | 4 | 0.26 |
| (condition * group) vs.  (condition * group * PCS) | 327.67  332.17 | 3.498 | 4 | 0.48 |
| (condition * group) vs.  (condition * group * FEDA_EV) | *327.67*  *323.68* | *11.991* | *4* | *0.02** |
| (condition * group) vs.  (condition * group * FEDA_AV) | 327.67  333.30 | 2.372 | 4 | 0.67 |
| (condition * group) vs.  (condition * group * FEDA_AM) | 327.67  334.94 | 0.730 | 4 | 0.95 |
| (condition * group) vs.  (condition * group * HIT-6)^a^ | 157.19  159.84 | 1.349 | 2 | 0.51 |
| (condition * group) vs.  (condition * group * pain days) | 318.24  323.29 | 2.951 | 4 | 0.57 |
| (condition * group) vs.  (condition * group * current clinical pain intensity) | 325.39  328.30 | 5.091 | 4 | 0.28 |
| (condition * group) vs.  (condition * group * average clinical pain intensity) | 324.05  325.85 | 6.195 | 4 | 0.19 |
| (condition * group) vs.  (condition * group * von Korff grade) | 324.05  327.03 | 5.020 | 4 | 0.29 |
|  |  |  |  |  |
| **Recollection (no pain – pain)** |  |  |  |  |
| (condition * group) vs.  (condition * group * fear) | *-89.82*  *-92.28* | *10.463* | *4* | *0.03** |
| (condition * group) vs.  (condition * group * expected interruption) | -90.78  -90.64 | 7.858 | 4 | 0.10 |
| (condition * group) vs.  (condition * group * PCS) | -92.28  -85.51 | 1.239 | 4 | 0.87 |
| (condition * group) vs.  (condition * group * FEDA_EV) | -92.28  -90.39 | 6.119 | 4 | 0.19 |
| (condition * group) vs.  (condition * group * FEDA_AV) | -92.28  -87.40 | 3.128 | 4 | 0.54 |
| (condition * group) vs.  (condition * group * FEDA_AM) | -92.28  -85.78 | 1.505 | 4 | 0.83 |
| (condition * group) vs.  (condition * group * HIT-6) ^a^ | -29.47  -25.94 | 0.468 | 2 | 0.79 |
| (condition * group) vs.  (condition * group * pain days) | -89.18  -88.70 | 7.528 | 4 | 0.11 |
| (condition * group) vs.  (condition * group * current clinical pain intensity) | -87.89  -84.67 | 4.779 | 4 | 0.31 |
| (condition * group) vs.  (condition * group * average clinical pain intensity) | -95.72  -90.19 | 2.472 | 4 | 0.65 |
| (condition * group) vs.  (condition * group * von Korff grade) | -95.72  -89.07 | 1.347 | 4 | 0.85 |

All linear mixed models are restricted to analyses involving the two patient groups only. ^a^ Model restricted to chronic migraine patients.

**P* < 0.05.

AIC, Akaike Information Criterion; FEDA-EV, Questionnaire of Experienced Attention Deficits - subscale fatigue and slowing down in practical activities; FEDA-AV, Questionnaire of Experienced Attention Deficits - subscale distractibility and slowing down in mental processes; FEDA-AM, Questionnaire of Experienced Attention Deficits - subscale reduction in motivation; HIT-6, Headache-Impact-Test; PCS, Pain Catastrophizing Scale.

**Supplementary Table 8 Stimulus intensities** **applied during the categorization task (first and final stimulus intensity; M ± SD)**

|  | **Group** | | |
| --- | --- | --- | --- |
| **Variable** | HC | CBP | CM |
| **First stimulus intensity (mA)** | | | |
| back | 2.28 ± 2.42 | 2.13 ± 2.25 | 1.84 ± 1.41 |
| head | 1.94 ± 2.25 | 1.86 ± 1.93 | 1.79 ± 1.81 |
| **Final stimulus intensity (mA)** | | | |
| back | 3.32 ± 2.99 | 3.36 ± 2.91 | 3.12 ± 2.19 |
| head | 3.21 ± 2.89 | 2.92 ± 2.21 | 2.88 ± 2.44 |

CBP, patients with chronic low back pain; CM, patients with chronic migraine; HC, Healthy controls; mA, milliamperes.

## **Supplementary References**

^1^ Helmstaedter C, Lendt M, Lux S. Verbaler Lern- Und Merkfähigkeitstest (VLMT). Hogrefe; 2001.

^2^ Härting Ch. Wechsler-Gedächtnistest - Revidierte Fassung: WMS-R; Manual ; Deutsche Adaptation Der Revidierten Fassung Der Wechsler Memory Scale. Huber; 2000.

^3^ Reitan RM. Validity of the trail making test as an indication of organic brain damage. Perceptual and Motor Skills. 1958;8:271-276.

^4^ Aschenbrenner S, Tucha O, Lange KW. Regensburger Wortflüssigkeits-Test (RWT). Hogrefe; 2000.

^5^ Zimmermann P, Fimm B. TAP. Testbatterie Zur Aufmerksamkeitsprüfung, Version 2.3. Psytest; 2013.
